# Liquid biopsy uncovers distinct patterns of DNA methylation and copy number changes in NSCLC patients with different EGFR-TKI resistant mutations

**DOI:** 10.1038/s41598-021-95985-6

**Published:** 2021-08-12

**Authors:** Hoai-Nghia Nguyen, Ngoc-Phuong Thi Cao, Thien-Chi Van Nguyen, Khang Nguyen Duy Le, Dat Thanh Nguyen, Quynh-Tho Thi Nguyen, Thai-Hoa Thi Nguyen, Chu Van Nguyen, Ha Thu Le, Mai-Lan Thi Nguyen, Trieu Vu Nguyen, Vu Uyen Tran, Bac An Luong, Linh Gia Hoang Le, Quoc Chuong Ho, Hong-Anh Thi Pham, Binh Thanh Vo, Luan Thanh Nguyen, Anh-Thu Huynh Dang, Sinh Duy Nguyen, Duc Minh Do, Thanh-Thuy Thi Do, Anh Vu Hoang, Kiet Truong Dinh, Minh-Duy Phan, Hoa Giang, Le Son Tran

**Affiliations:** 1 University of Medicine and Pharmacy at Ho Chi Minh City, Ho Chi Minh City, Vietnam; 2 Medical Genetics Institute, Ho Chi Minh City, Vietnam; 3 Vietnam National Cancer Hospital, Ha Noi, Vietnam; 4 Ha Noi Oncology Hospital, Ha Noi, Vietnam; 5 Thu Duc District Hospital, Ho Chi Minh City, Vietnam; 6 FV Hospital, Ho Chi Minh City, Vietnam

**Keywords:** Cancer, Genetics, Molecular biology, Biomarkers, Molecular medicine, Oncology

## Abstract

Targeted therapy with tyrosine kinase inhibitors (TKI) provides survival benefits to a majority of patients with non-small cell lung cancer (NSCLC). However, resistance to TKI almost always develops after treatment. Although genetic and epigenetic alterations have each been shown to drive resistance to TKI in cell line models, clinical evidence for their contribution in the acquisition of resistance remains limited. Here, we employed liquid biopsy for simultaneous analysis of genetic and epigenetic changes in 122 Vietnamese NSCLC patients undergoing TKI therapy and displaying acquired resistance. We detected multiple profiles of resistance mutations in 51 patients (41.8%). Of those, genetic alterations in *EGFR*, particularly *EGFR* amplification (n = 6), showed pronounced genome instability and genome-wide hypomethylation. Interestingly, the level of hypomethylation was associated with the duration of response to TKI treatment. We also detected hypermethylation in regulatory regions of Homeobox genes which are known to be involved in tumor differentiation. In contrast, such changes were not observed in cases with *MET* (n = 4) and *HER2* (n = 4) amplification. Thus, our study showed that liquid biopsy could provide important insights into the heterogeneity of TKI resistance mechanisms in NSCLC patients, providing essential information for prediction of resistance and selection of subsequent treatment.

## Introduction

Lung cancer is the most common type of cancer and the leading cause of cancer related deaths globally, of which the majority of cases are non-small cell lung cancer (NSCLC)^1,2^. Clinical studies have shown that patients carrying sensitizing *EGFR* mutations displayed prolonged progression-free survival upon receiving first generation tyrosine kinase inhibitors (TKI) including erlotinib and gefitinib^3–5^ or the second generation TKI afatinib^6,7^. Such TKI drugs were shown to either reversibly (first generation TKI, 1G TKI) or irreversibly (second generation TKI, 2G TKI) bind to the ATP binding site of the tyrosine kinase domain of *EGFR*, thereby inactivate its activity^8^. Both 1G and 2G TKI drugs have been proposed as the first line treatment for patients with advanced NSCLC who carry TKI targeted mutations^9^.

Despite the superiority of 1G and 2G TKIs over chemotherapy as first line treatment, almost all patients develop resistance to TKI and show disease progression after a median of 10–14 months of TKI therapy^10^. Resistance to TKI drugs could either stem from intrinsic or acquired resistance^11^. Patients with intrinsic resistance fail to respond to initial treatment within less than 3 months^12,13^. This type of resistance could be due to genetic alterations in driver genes that already exist in tumor cells and confer resistance to therapy^14^. In contrast, according to the criteria from the World Health Organization (WHO) or Response Evaluation Criteria in Solid Tumours (RECIST), acquired resistance responses are related to secondary genetic alterations that emerge after a complete or partial response to targeted therapies or more than 6 months of stable disease, resulting in tumor survival and continued growth^15,16^. NSCLC patients could develop a secondary EGFR substitution of threonine to methionine at position 790 (T790M) in exon 20 or *EGFR *amplification at the time of acquired resistance to erlotinib or gefitinib. In addition to *EGFR* dependent resistance mechanisms, the activation of bypass pathways through the MET, AXL, IGF1R axis or the phenotypic transformation to small-cell lung cancer (SCLC) has been reported to drive resistant responses to 1G and 2G TKIs^17–20^.

Although genetic alterations are known to be associated with TKI resistance, it has become increasingly evident that multiple mechanisms could contribute to the complex process of resistance development^21^. Epigenetic alterations, particularly DNA methylation, have been reported to affect the sensitivity of cancer cells to TKI drugs through their broad impact on regulation of gene expression^22–24^. Hypermethylation at promoter regions of certain tumor suppressor genes (TSG) was associated with increased resistance to gefitinib in lung cancer cell lines through silencing their expression^25^. Moreover, global hypomethylation has recently been proposed as a hallmark of cancer, which tends to occur in promoter regions of oncogenes, leading to activation of these genes^26,27^. It is thought that the abnormal hypomethylation at genome-wide level is linked with copy number aberration (CNA), resulting in genome instability, an important tumorigenic event^28,29^. How methylation changes are linked with genetic mutations during the acquisition of TKI resistance remains unknown. Most current knowledge of TKI resistance mechanisms is based on in vitro cell line models that focus on elucidating the contribution of either mutation or methylation changes of specific genes. As such, findings from such models may under-represent the complexity of resistance mechanisms in NSCLC patients.

In this study, we examined the application of plasma cell free DNA (cfDNA) to simultaneously profile genetic and methylation landscapes in 122 Vietnamese NSCLC patients who developed acquired resistance to TKI drugs. Our study showed that genetic mutations, genome-wide and hotspot methylation states, and genome instability are important signals associated with acquired resistance to TKI drugs. The combination of these signals provides essential information for prediction of resistance and selection of subsequent treatment.

## Results

### Clinical features of patient cohort

In this study, a total of 122 patients with advanced NSCLC were recruited from three hospitals in Vietnam (Vietnam National Cancer Hospital, Hanoi Oncology Hospital and Thu Duc District Hospital). The majority were treated with first generation TKI erlotinib (82 cases, 67.2%) and gefitinib (14 cases, 11.5%) while 3 patients (2.5%) received second generation TKI afatinib as first-line treatment (Table 1). Five patients were given erlotinib as an initial treatment, followed by a second-line treatment with either gefitinib (2 cases, 1.6%) or afatinib (3 cases, 2.5%). The remaining patients (18 cases, 14.8%) were confirmed to be administered with either first or second generation TKI but the specific drug name was not recorded (Table 1). Liquid biopsy from all 122 patients were sampled at the time of disease progression after achieving partial or stable disease (> 3 months) or at least 6 months post TKI treatment, satisfying the criteria of acquired resistance proposed by RECIST (Response Evaluation Criteria in Solid Tumours) or WHO^15,16^. The majority of patients were diagnosed at advanced stages (III-IV) (82%) with metastatic adenocarcinoma cancer (69.7%) (Table 1). Additional clinical characteristics and complete mutation results of all 122 patients are summarized in Table S1.

**Table 1:** Clinical characteristics of 122 Vietnamese NSCLC with acquired resistance to TKI drugs.

Clinical characteristics	N	%
**Sex**		
Female	71	58.2
Male	48	39.3
Unknown	3	2.5
**Age**		
Median	61	
Min	29	
Max	77	
Unknown	2	
**Histology**		
AC	85	69.7
SCC	4	3.3
Unknown	33	27
**Tumor stage**		
III-IV	100	82
Unknown	22	18
**Smoking**		
Yes	15	12.3
No	33	27
Unknown	74	60.7
**TKI treatment regimen**		
Erlotinib	82	67.2
Gefitinib	14	11.5
Erlotinib	2	1.6
+ Gefitinib		
Afatinib	3	2.5
Erlotinib	3	2.5
+ Afatinib		
Unknown TKI	18	14.8

*N* total case number, *%* percentage of cases.

### Heterogeneous mutational profiles of NSCLC patients undergoing acquired resistance to 1G or 2G TKI drugs

**Figure 1:**
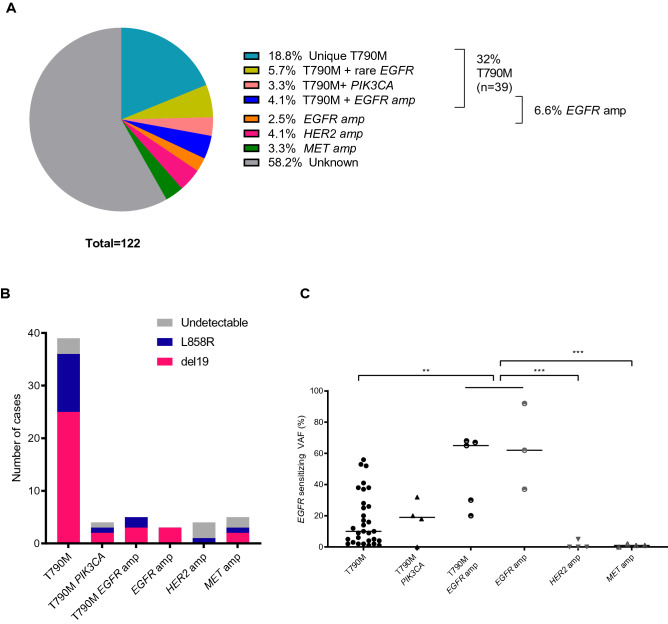
The heterogeneity of mutation profiles of 122 Vietnamese NSCLC patients with acquired resistance to first and second generation EGFR TKI. (**A**) Distribution of TKI resistance associated mutations of 122 Vietnamese NSCLC. (**B**) Frequencies of *EGFR* sensitizing mutations (del19, L858R, or rare *EGFR* mutations) in different resistance mutation profiles. (**C**) Variant allele frequency (VAF, %) of *EGFR* sensitizing mutations in different resistance mutation profiles. Data are presented as median VAF, each data point represents one patient. **p < 0.01; ***p < 0.001 (one-way ANOVA, Kruskal–Wallis test).

To profile the genetic alterations associated with TKI resistance, we used a liquid biopsy based assay to identify mutations, either single base substitutions or amplifications, in 9 major driver genes including *EGFR*, *KRAS*, *BRAF*, *NRAS*, *ALK*, *ROS1*, *PIK3CA*, *MET* and *HER2*. We detected multiple mutations that could potentially drive acquired resistance responses to TKI drugs in 51 patients (41.8%) (Fig. 1A).

Of the known resistance mutations, *EGFR* T790M was detected in 39 cases (32%), representing the most common resistance mutation among the Vietnamese NSCLC patients (Fig. 1A). Of those cases, the majority (23 cases, 18.8%) carried only the T790M mutation while the remaining 16 cases (13.2%) harbored extra co-occurring mutations. Seven of them (5.7%) carried a variety of rare *EGFR* mutations that were defined as non-classical mutations in the tyrosine kinase-domain (exons 18–21) including S768I (1 case), co-occurring S768I and G719C (2 cases), A750P (2 cases), V834L (1 case), and E709K (1 case). In addition, all 4 cases (2.5%) with *PIK3CA* mutations including 3 with mutation in codon 545 (E545K) and 1 with mutation in codon 1047 (H1047R) co-occurred with T790M (Figs. 1A and S1). The co-occurrence of T790M and *EGFR* amplification was detected in 5 (4.1%) out of 8 cases with *EGFR* amplification (Figs. 1A and S1).

In addition to mutations in *EGFR*, we detected resistance variants in off target genes including *MET* and *HER2* amplification in 4 (3.3%) and 5 cases (4.1%), respectively (Fig. 1A). *MET* and *HER2* amplification mutations appeared to be mutually exclusive with *EGFR* mutations (Figs. 1A and S1). No mutation was detected in *KRAS*, *NRAS*, *BRAF*, *ALK* or *ROS1*.

At the time of acquired resistance, the primary sensitizing mutations, either del19 or L858R were retained in almost all cases (46/51, 90.2%) carrying known resistance mutations, except for those with *HER2* and *MET* applications showing either complete loss of sensitizing mutations or markedly lower VAFs (ranging from 1 to 2%) than cases with mutations in *EGFR* (Fig. 1B,C). By contrast, cases with *EGFR* amplification or those with co-occurrence of T790M and *EGFR* amplification harbored significantly higher sensitizing mutation VAFs than other cases without amplification (p < 0.01, Fig. 1C). We further found a significant positive correlation between levels of *EGFR* amplification and sensitizing VAFs (r = 0.94, 95% CI 0.68–0.99, p = 0.0006, Fig. S2A). These results suggested that *EGFR* amplification might occur in the primary *EGFR* sensitizing clones while *HER2* and *MET* amplification could arise from de novo resistance tumor clones. For the majority of T790M + cases (33/39 cases, 84.6%), T790M had VAF lower than their corresponding sensitizing mutations (Fig. S2B), indicating that T790M might be acquired by resistance sub-clones.

Taken together, the analysis of genetic alterations in plasma samples revealed both inter- and intra-tumor heterogeneity of mutational profiles in Vietnamese NSCLC patients with acquired resistance to TKI drugs.

### Distinct genome-wide methylation and copy number alterations in patients with EGFR dependent and independent resistance mechanisms

**Figure 2:**
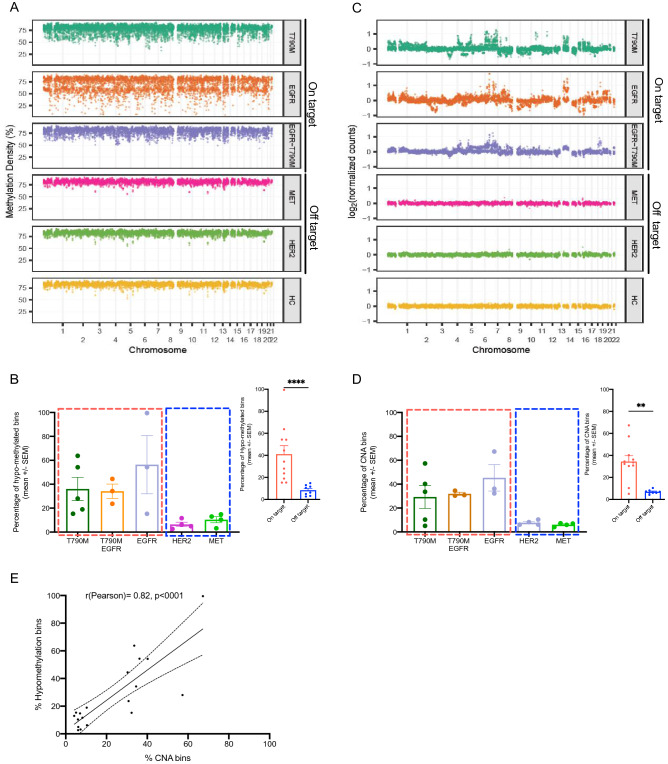
EGFR dependent (on-target) and independent (off-target) resistance mechanisms showed distinct landscapes of genome-wide methylation and copy number changes. (**A**, **C**) Methylation density (MD) (**A**) and DNA copy number (**C**) alterations for each 1 Mb bin along the chromosomes (chr1-22). Cases with T790M (n = 5), *EGFR* amplification (n = 3) and T790M and *EGFR* amplification co-occurrence (n = 3) were grouped into the on-target group while *HER2* amplification (n = 4) and *MET* amplification (n = 4) cases were grouped into the off-target group. Healthy individuals (n = 20) were used as a reference group to determine the baseline levels of MD and CNA. (**B**, **D**) Percent of hypo-methylated bins (**B**) and CNA bins (**D**) in cases with different resistance mutation profiles grouped into the on-target group or the off-target group. The component bar graphs showed the comparison between the two groups. Data are shown as mean ± SEM. **p < 0.01, ****p < 0.0001 (Mann–Whitney test). (**E**) Linear regression and Pearson’s correlation between proportion of hypo-methylated bins and CNA bins in 19 patients from both on-target and off-target groups.

Genome-wide epigenetics, particularly DNA methylation changes, have been implicated in the acquisition of drug resistance by cancer cells^30^. However, the relationship between DNA methylation and mutation alterations in mediating resistance to TKI drugs remains largely unknown. To profile the methylation changes at the genome-wide level of different resistance mutation groups, we employed the workflow previously reported by Jiang et al.^31^. The assay was based on the combination of bisulfite conversion and massively parallel sequencing to analyze methylation density (MD) in bins of 1 Mb across 22 chromosomes. The analysis was performed for all cases with *EGFR* amplification (3 cases) and MET amplification (4 cases). For the remaining cases, only 3 out of 5 cases with co-occurring T790M and *EGFR* amplification and 4 out of 5 cases with *HER2* amplification had sufficient cell free DNA for the analysis. Among 23 patients carrying T790M mutation, we randomly selected 5 cases with sufficient plasma. The total 19 chosen cases were grouped into either EGFR dependent resistance mutations (on-target) or EGFR independent mutations (off-target) which have comparable median age (69 versus 64.5 years, p > 0.05) (Fig. S3A). Strikingly, cases with resistance mutations in *EGFR* including T790M only, *EGFR* amplification and co-occurrence of *EGFR* amplification and T790M displayed extensive reduction in methylation density at genome-wide level compared to that of healthy subjects. By contrast, cases with off-target resistance mutations including *HER2* and *MET* amplification showed comparable methylation density to healthy control subjects (Fig. 2A). We further quantified the proportion of hypo-methylated bins that have the MD lower than 3 SDs below the mean of the corresponding bins of the healthy subjects. We found a significantly higher proportion of hypo-methylated bins in cases with on target resistance mutations compared to those with off-target mutations (38.9% versus 8.3%, p < 0.01, Fig. 2B). There was no statistically significant difference in the proportion of hypo-methylated bins among the on-target mutation groups (Fig. 2B).

Genome-wide hypo-methylation has been associated with genome instability in cancer cells as reflected in DNA copy number aberrations (CNA)^28^. Hence, we further explored CNA in different resistance mutation groups. Consistent with the trend of hypo-methylation, we detected pronounced alterations in DNA copy number at multiple regions in cases with on-target resistance mutations, which were not observed in those carrying off-target mutations (Fig. 2C,D). As expected, we observed a significant correlation between numbers of hypo-methylated bins and CNA bins in all tested cases (R = 0.82, 95% CI 0.60–0.93%, p < 0.0001, Fig. 2E) and such alterations tended to occur across 22 chromosomes instead of localizing to any particular chromosomes (Fig. S3B,C).

These results showed that cases with on-target resistance mutations carried remarkable genome-wide hypo-methylation and CNA while not much changes were detected in off-target resistance mutation groups.

### Genome-wide hypo-methylation and CNA correlated with the duration of drug response in EGFR amplification cases

**Figure 3:**
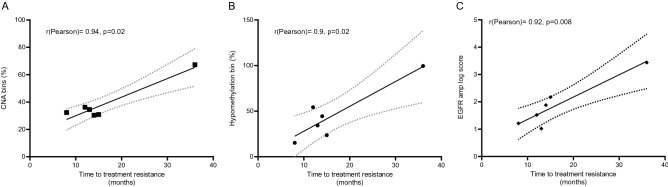
Accumulation of genetic and epigenetic changes in cases with EGFR amplification correlated with the duration of response to TKI treatment. (**A**–**C**): Linear regression and Pearson’s correlation between proportion of CNA bins (**A**), hypo-methylated bins (**B**), EGFR amplification scores (**C**) and time to treatment resistance (TTTR) in 6 cases with *EGFR* amplification including 3 cases with co-occurring T790M.

The extensive changes in global hypo-methylation and CNA detected in cases with on-target mutations could be accumulated during the response to TKI drug treatment and may play important roles in the acquisition of resistance. Thus, we examined the correlation between these changes and the time to treatment resistance (TTTR). Since the levels of hypo-methylation and CNA were similar between *EGFR* amplification cases and those carrying co-occurring T790M and *EGFR* amplification, we put these samples into the same *EGFR* amplification group for the correlation analysis. Although both *EGFR* amplification and T790M cases displayed extensive changes in hypo-methylation and CNA, only *EGFR* amplification cases showed significantly positive correlations between CNA or hypo-methylation changes with TTTR (r = 0.94, 95% CI 0.6–0.99, p = 0.004 for CNA, Fig. 3A; r = 0.9 95% CI 0.3–0.98, p = 0.02 for hypo-methylation, Fig. 3B; insignificant correlations, p > 0.05 in T790M cases, Fig. S4A,B). Moreover, we found that the abundance of *EGFR* amplification mutations but not T790M in plasma significantly correlated with TTTR (r = 0.92, 95% CI: 0.5–0.99, p = 0.008, Fig. 3C for *EGFR* amplification cases and r = -0.18, p > 0.05; insignificant correlation, p > 0.05 for T790M cases, Fig. S4C). These results suggested that changes in DNA hypo-methylation and CNA concurrently occurred with *EGFR* amplification and were gradually accumulated during the treatment with TKI drugs. This process may be required to ensure the survival of tumor cells before acquiring the resistance.

### Distinct hyper-methylation of transcriptional regulatory regions of cancer related genes in patients with EGFR dependent and independent resistance mutations

**Figure 4:**
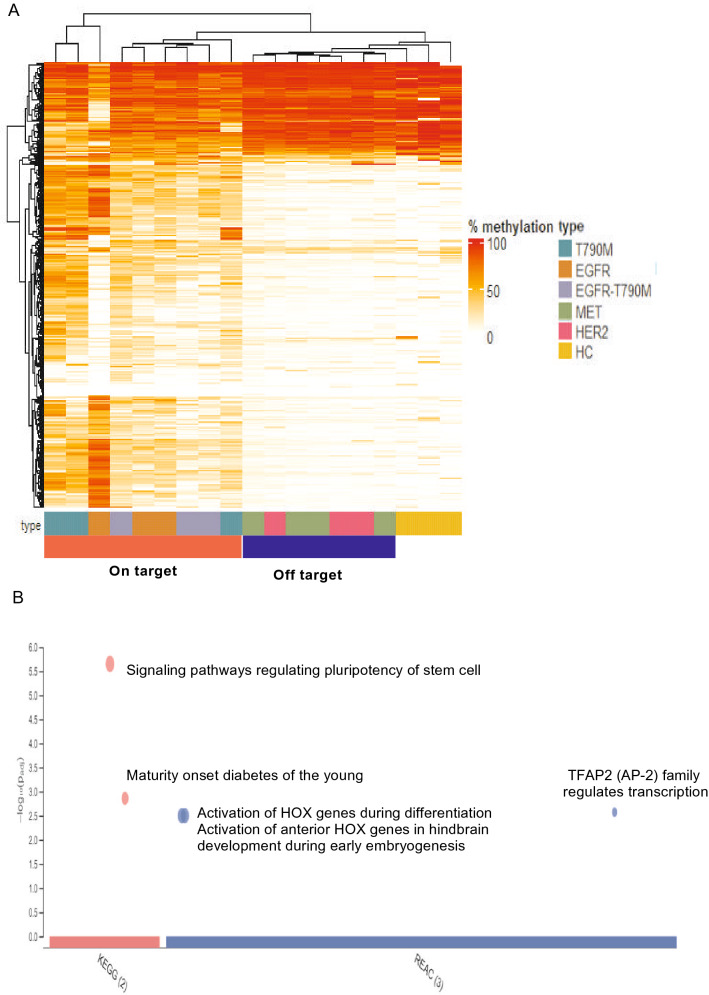
EGFR dependent resistance mechanisms displayed markedly high levels of hyper-methylation in the transcriptional regions of genes involved in regulation of differentiation. (**A**) Heat-map showed differentially methylated regions (DMRs) among 450 target regions mapped to regulatory regions of cancer related genes across samples within the on-target group (T790M, T790M-EGFR amplification and EGFR amplification) and off-target group (HER2 and MET amplification). Healthy subjects were used as a reference group to determine the basal levels of methylation at target regions. Heatmap visualization was analyzed with ComplexHeatmap package version 2.8.0 (https://www.bioconductor.org/packages/release/bioc/html/ComplexHeatmap.html). Hierarchical clustering was done using Euclidean distance. Red in the scale bar represents a high level of methylation. (**B**) KEGG and Reactome pathway enrichment analysis using g: Profiler (https://biit.cs.ut.ee/gprofiler) for genes associated with 202 DMRs showing hyper-methylation in the on-target group compared to the off-target group.

The acquisition of resistance to TKI might be associated with changes in expression of many genes, which could be affected by the methylation states of their transcriptional regulatory regions^23,24^. To identify such genes, we designed a panel of capture probes targeting 450 regions (Table S2) which were selected based on their significant contributions to cancer carcinogenesis reported by previous publications^32–37^. The majority of regions contain CpG islands overlapping or located in close proximity to promoter regions of cancer related genes including oncogenes and tumor suppressor genes. Distinct methylation patterns were observed in cases with on-target resistance mutations compared to those carrying off-target mutations, which showed methylation profiles similar to healthy control subjects (Fig. 4A). A total of 260 differentially methylated regions (DMR) out of 450 regions (57.8%) were identified when comparing on-target and off-target mutation groups. Of those, the majority (202 DMR) were significantly hyper-methylated (log_2_FC > 1, FDR < 0.05, Table S3) while the remaining 58 DMR were found hypo-methylated in cases with on-target resistance mutations compared to those with off-target mutations. Pathway enrichment analysis by gProfiler was performed on the set of genes mapped by those DMR. We found that 202 hyper-methylated regions were enriched in 5 pathways from the REACTOME and KEGG database including pathways regulating activation of HOX genes (2 pathways), the transcriptional regulation by TFAP2, the pluripotency of stem cells and the maturity of onset diabetes of the young (Fig. 4B). The sets of genes associated with those pathways were listed in Table S4. The activation of HOX genes and the regulation of transcription by TFAP2 pathways consisted of several component genes that were tumor suppressor genes involved in the regulation of tumor differentiation. The hyper-methylation at regulatory regions of such genes in cases with on-target resistance mutations could lead to the suppression of their expression, resulting in tumor progression. The functional contribution of the remaining two pathways to cancer tumorigenesis is not well understood. There was no significant pathway enriched by the 58 hypo-methylated DMR. The majority of those DMR were found to be fully methylated in cases with *MET* and *HER2* amplification as well as healthy individuals, suggesting the re-activation of such genes in the on-target groups via reduced methylation at their promoter regions. These results showed that cases carrying resistance mutations in *EGFR* accumulated abundant hyper-methylation changes in regulatory regions of tumor suppressor genes such as HOX genes to inhibit the differentiation of cancer cells during the acquisition of TKI resistance. By contrast, cases with HER2 and MET amplification developed resistance to TKI via mechanisms independent of the acquisition of hyper-methylation of such genes.

## Discussion

Acquiring resistance to TKI drugs is a complex and dynamic process possibly governed by both genetic and epigenetic changes^38^. Profiling mutation and methylation landscapes simultaneously could lead to comprehensive understanding of the mechanisms for acquiring resistance and providing essential information for designing secondary treatment to overcome resistance. In patients with advanced NSCLC, liquid biopsy has become the only ethically feasible method for sampling and profiling these changes, highlighting its role in both academic research and clinical application. Here we took advantage of liquid biopsy to sample a large number of patients, and used ctDNA extracted from their blood to detect both genetic and epigenetic changes by massively parallel sequencing.

We uncovered the heterogeneity of both mutation and methylation alterations in 122 Vietnamese NSCLC patients who developed acquired resistance to 1G and 2G TKI drugs (Fig. 1). Multiple resistance mutations were detected in 51/122 patients (41.8%). Of those, T790M was identified as being the most frequent resistance mutation, detected in 32% of all cases, which is consistent with previous studies reporting a range of 30% to 50% from ctDNA^39,40^. In addition to mutations in the on-target gene *EGFR*, we detected substitution mutations in *PIK3CA* (E545K and H1047R) and amplification of *MET* and *HER2*, which have been reported to activate the bypass pathways of *EGFR* signaling^41,42^. These findings suggested that multiple resistance mutations could be associated with acquired resistance to TKI drugs, highlighting the inter-individual heterogeneity of resistance mechanisms. Our analysis also revealed the intra-tumor heterogeneity of resistance mutation profiles. Indeed, we detected multiple co-occurring resistance mutations present in the background of tumors carrying *EGFR* mutations in our cohort. It is thought that a single substitution in T790 codon, a key position in the ATP binding pocket, is sufficient to drive acquired resistance to first generation TKIs^43^. However, the co-occurrence of multiple resistance mutations in individual patients has been reported to be responsible for the failure of treatment targeting a single driver mutation in heterogeneous tumors^41^. This point was clearly demonstrated in our data where 33.3% (13/39) of T790M positive cases co-existed with other resistance-associated mutations including *EGFR* amplification and *PIK3CA* mutations. In addition, we detected a variety of rare *EGFR* mutations in T790M positive cases including mutations in codon E709, G719, A750, S768, V834L and L861 in 10 cases, with 8/10 cases also carrying T790M. Although these rare *EGFR* mutations are known to drive conformational changes in the drug binding site of EGFR, their contributions to TKI drugs responses are still under debate and warrant future clinical validation^44^.

We observed that the majority of cases carrying EGFR on-target mutations retained the primary sensitizing mutations, either del19 or L858R at the time of resistance. This suggested that EGFR on-target resistance mutations might arise from the same primary tumor clones. Among those cases, *EGFR* amplification cases displayed remarkably high levels of sensitizing VAFs which were significantly correlated with amplification scores (Fig. 3C), suggesting that the amplification occurred in the primary sensitizing clones and affected the copy number of the allele containing sensitizing *EGFR* mutations. In contrast to the on-target mutation cases, those with *MET* or *HER2* amplification showed loss of primary sensitizing mutations and did not carry any other known resistance mutations. This suggested that *MET* and *HER2* amplification could be de novo mutations and present as the sole drivers of resistance to TKI drugs.

Interestingly, our global methylation investigation also revealed the heterogeneity of methylation profiles in cases harboring different resistance mutation profiles. Cases with on-target mutations including T790M, *EGFR* amplification and their co-occurrence showed extensive hypo-methylation and CNA at genome-wide level which were not detected in off-target mutations including MET and HER2. This highlighted that different resistance mutation profiles were associated with different methylation changes. The interrelationship between DNA methylation and genetic changes is complex and possibly bidirectional^21,45^. It remains unknown whether mutations and methylation changes have causative roles or bidirectional relationship^45^.

We further detected the correlations between the levels of hypo-methylation or CNA and the duration of drug response for cases with *EGFR* amplification. Such correlations suggested that the amplification of *EGFR* and genome instability might have occurred concurrently and that they were accumulated gradually and possibly required for tumor cells to sustain their growth under the pressure of drug selection. In support to this notion, a previous study using both in vitro and in vivo models showed that amplification of the wild-type *EGFR* allele conferred resistance to TKI drugs through alleviating the inhibitor pressure of TKI drugs^46^. In contrast, T790M cases did not show correlation with the duration of drug response, suggesting that the changes in hypo-methylation and CNA in those cases could be passenger events that might not have a functional role during the response to 1G or 2G TKIs. Our finding was consistent with a recent study showing that genome-wide CNA was detected in patients carrying T790M at the time of resistance to gefitinib and that the presence of CNA was associated with poor response to subsequent treatment with 3G TKI, osimertinib^47^. *MET* and *HER2* amplification did not show noticeable changes in both global methylation and CNA, suggesting that the acquisition of genome instability during the response to TKI drugs might be unique to the EGFR dependent resistance mechanisms.

In addition to changes at genome-wide levels, we also detected hyper-methylation of regulatory regions of multiple genes that might be involved in TKI resistance. Of those, a group of Homeobox (HOX) genes involved in regulating growth and differentiation of cancer cells were found to be hyper-methylated. The hyper-methylation status at transcriptional regulatory regions of those genes indicated their expression was suppressed in cases with EGFR dependent resistance mutations. Our finding was consistent with a recent study by Shu et al.^48^ showing that increased methylation of *HOXB9* correlated with higher rate of resistance to EGFR-TKI. Thus, such consistent findings indicated that the hyper-methylation status of HOX genes could be exploited to predict and treat *EGFR* amplification mediated resistance.

There were several limitations in our study. First, this was a retrospective collection of cases for which the cumulative changes of genetic and methylation information were analysed at the time of TKI resistance. Therefore, our analysis was unable to elucidate how mutation or methylation changes evolved during the treatment process. A longitudinal analysis is warranted to understand the relationship of such changes. Second, a main limitation of our study is the small sample size. Due to low frequencies of certain mutation subtypes and insufficient plasma samples, only a small number of samples in each group were subjected to methylation and CNA analysis. Thus, our current study might be considered as exploratory analyses and future studies with a larger cohort are required for robust analysis of resistance mechanisms among different mutation profiles. Third, due to the lack of paired tumor biopsies, the amplifications detected by our liquid biopsy method could not be validated by a gold standard technique such as FISH. However, the higher VAF of sensitizing mutation or T790M in cases with *EGFR* amplification compared to those without *EGFR* amplification suggested that the amplification events detected were true positives. Lastly, we could not detect any known resistance mechanisms for 71 patients (58.2%) in our cohort, suggesting that there were additional mechanisms driving acquired resistance and not captured by our assay. The additional mechanisms could include changes in tumor histology with tumor cells displaying features of small-cell lung cancer (SCLC) or epithelial mesenchymal transition (EMT), or alterations in other novel genes involved in the MAP kinase and mTOR pathway^41^. Therefore, an extended panel covering additional driver genes or higher resolution of methylation and CNA analysis will be required to identify new resistance mechanisms.

In conclusion, we have shown a clinical application of liquid biopsy in profiling both mutation and methylation alterations in patients with advanced NSCLC who developed resistance to TKI drugs. Our findings uncovered distinct patterns of DNA methylation and copy number changes between patients harboring either *EGFR* dependent and independent resistance mutations (Fig. S5), highlighting the heterogeneity of acquired resistance to TKI drugs for which several pathways may be involved.

## Methods

### Patient recruitment

A total of 122 patients from Vietnam National Cancer hospital, Ha Noi Oncology hospital and Thu Duc district hospital were recruited to this study between 2018 and 2020. 54 out of 122 patients were from the research funded by Vietnam National Foundation for Science and Technology Development (NAFOSTED). The recruitment criteria are patients with advanced NSCLC (stage III or IV) who had received either first (gefitinib or erlotinib) or second generation (afatinib) EGFR-TKI therapy and showed acquired resistance responses (according to WHO or RECIST criteria)^16,49^. We also recruited 20 healthy individuals to obtain cfDNA for validation of our MET amplification detection pipeline. Plasma samples were collected at the time of disease progression and after at least 6 months following initial TKI drug treatment. This study was approved by the Ethic Committee of University of Medicine and Pharmacy at Ho Chi Minh City, Vietnam. All patients and healthy individuals were given written informed consent prior to participation in the study. All methods were performed in accordance with the relevant guidelines and regulations.

### Plasma cell free DNA isolation

Peripheral blood (10 mL) was drawn in Streck tubes (Cell-free DNA BCT, Streck) and subject to two rounds of centrifugation (2000×*g* for 10 min then 16,000×*g* for 10 min) to separate plasma from blood cells. The plasma fraction (4–6 mL) was collected, aliquoted (2 mL per aliquot) and stored at − 80 °C until cell free DNA extraction.

Cell free DNA was extracted from an aliquot of 2 mL of plasma using the MagMAX Cell-Free DNA Isolation kit (Thermo Fisher, USA) following the manufacturer’s instructions.

### Ultra-deep massively parallel sequencing (MPS) with unique molecular identifier tagging

Cell free DNA was prepared and sequenced as previously described^50^. Briefly, a library with unique molecular identifier tagging was prepared from 2 ng of cfDNA using the Accel-NGS 2S Plus DNA library kit (Swift Biosciences, USA) following the manufacturer's instructions. Library concentrations were quantified using QuantiFluor dsDNA system (Promega, USA). Equal amounts of libraries (150 ng per sample) were pooled together and hybridized with xGen Lockdown probes for 9 targeted genes *EGFR*, *KRAS*, *NRAS*, *BRAF*, *ALK*, *ROS1*, *MET*, *HER2* and *PIK3CA* (IDT DNA, USA). For *ALK* and *ROS1*, customized probes for intron regions were designed and mixed with probes for exon regions at equal concentrations. For detection of plasma *EGFR*, *HER2* and *MET* amplification, circulating tumor DNA copy number variation pipeline was used as previously described^51^. The pipeline was evaluated using cfDNA sequencing data of 20 healthy people and positive MET amplification control DNA (Horizon, USA).

Sequencing was run using NextSeq 500/550 High output kits v2 (150 cycles) on Illumina NextSeq 550 system (Illumina, USA) with minimum target coverage of 10,000 ×. A total on-target fraction of 20–40% was detected.

### Variant calling using Mutect2

Each sample was barcoded with a single 8-bp index in the P7 primer and each DNA fragment was tagged with a unique identifier consisting of a random 9-bp sequence within the P5 primer. Pair-end (PE) reads and the corresponding unique identifier sequences were generated using the bcl2fastq package (Illumina). The reads were aligned to the human genome (hg38) using BWA and then grouped by the unique identifier in order to determine a consensus sequence for each fragment, eliminating sequencing and PCR errors that account for less than 50% of reads per fragment. The consensus reads were used for final variant calling using Mutect2^52^. A custom pipeline with call to BWA 0.7.1^53^, Picard 2.18.23 (Broad Institute, GitHub Repository), Samtools 1.9^54^ and Fulcrum genomic analysis packages^51^ were built to perform the above-mentioned analysis steps. Unique molecular identifier (UMI) grouping was performed by using the fgbio 0.8.0 package (Fulcrum Genomics)^51^. For detection of *ALK* and *ROS1* rearrangement, fusion variant calling was analyzed using Factera v1.4.4 with default parameters^54^. For detection of *EGFR*, *MET* and *HER2* amplification, FACETS V0.5.6 was used to analyse copy number variation^55^.

### Bisulfite treatment and sequencing library preparation

Among cases with known mutational profiles, 19 cases had sufficient extracted cell free DNA were analysed for methylation changes at both genome-wide and targeted regional levels. A minimum of 2 ng isolated cfDNA of those samples was subjected to bisulfite conversion using EZ DNA Methylation-Gold™ Kit (ZYMO RESEARCH, D5006, USA). Bisulfite-converted DNA was subject to dual indexed library preparation using Accel-NGS® Methyl-Seq DNA Library Kit (Swift Biosciences, 30024, USA) and the library products were subsequently divided into 2 portions for target and genome wide sequencing.

### Genome wide methylation and CNA analysis

One half of the DNA library was directly sequenced on MGI DNBSEQ-G400 system in a 100 bp paired end format and at a sequencing depth of 20 million reads per case (equivalent to 0.6 ×). The analysis of methylation and CNA was performed using the Methy-Pipe^31^. Briefly, the genome was divided into multiple 1 Mb bins. The methylation density (MD) for each bin was calculated as the number of methylated cytosines in the context of CpG dinucleotides divided by the total number of cytosines at CpG positions. Hypomethylated bins are defined as those with MD lower than 3 SDs (standard deviation) below the mean of the corresponding bin of the healthy subjects. For CNA analysis, sequenced reads aligned to each bin were counted. In order to prevent GC bias, sequence counts were corrected by GC-content and normalized with the median read counts of all bins (QDNAseq)^56^. The z-score of each bin was then calculated based on the mean and SD of the corresponding bin in the healthy subjects. The bin with aberration in copy number was defined as having the z-score > 3 or < − 3. Subsequently, the proportion of CNA bins for each sample was calculated.

### Target methylation analysis

Half of the DNA library was enriched using a customized panel of xGen Lockdown Probes targeting 450 regions and covering 9593 CpG sites (IDT, USA). The panel was constructed based on previous publications and the 450 regions were mapped to regulatory regions including promoters or enhancers of tumor suppressor genes involving cancer progression (Table S2)^32–37^. The hybridization was performed using XGen hybridization and wash kit (IDT, 1072281, USA). Each sample was sequenced at 20 million reads on the MGI DNBSEQ-G400 system. After trimming sequencing adapters, sequenced reads were aligned to reference genome and methylation calling was performed using Bismark aligner^57^. The methylation patterns were analyzed to find the differences among sample groups based on the methylation percentage of each region examined. The methylation percentage of each region is calculated by the formula: %Methylation = methylated cytosine (C)/(methylated C + un-methylated C). The methylated cytosines were those cytosines recovered among the CpG dinucleotides on the sequenced reads mapped to the target regions whereas un-methylated C were recovered thymine among the CpG dinucleotides on the sequenced reads mapped to the target regions.

### Statistical analysis

Wilcoxon Rank Sum test and was performed for identifying the regions that were differentially methylated between EGFR dependent and independent groups. The p values were corrected by the Benjamini Hochberg method for multiple comparisons. Pearson’s correlation analysis was performed to analyze the correlations between levels of hypo-methylation or CNA and time to treatment resistance or the correlation of mutation abundance between the resistance and sensitizing mutations. Pathway analysis was carried out using gProfiler to identify biological pathways significantly enriched based on the set of genes associated with DMR^58^.

## Supplementary Information

Supplementary Legends.

Supplementary Figure S1.

Supplementary Figure S2.

Supplementary Figure S3.

Supplementary Figure S4.

Supplementary Figure S5.

Supplementary Table S1.

Supplementary Table S2.

Supplementary Table S3.

Supplementary Table S4.

## Data Availability

All data generated or analyzed during this study are included in this published article. Sequencing data will be deposited in a public portal database (NCBI SRA) upon acceptance.
