# Supplementary material for: Liquid biopsy uncovers distinct patterns of DNA methylation and copy number changes in NSCLC patients with different EGFR-TKI resistant mutations
Source: Sci Rep. 2021 Aug 12;11:16436. doi: 10.1038/s41598-021-95985-6 (PMC8361064; doi:10.1038/s41598-021-95985-6)
Supplement: Supplementary file 1 — Supplementary Legends. [file 41598_2021_95985_MOESM1_ESM.pdf]

Liquid biopsy uncovers distinct patterns of DNA methylation and copy number changes in NSCLC patients with different EGFR-TKI resistant mutations

Hoai-Nghia Nguyen, Ngoc-Phuong Thi Cao, Thien-Chi Van Nguyen, Khang Nguyen Duy Le, Dat Thanh Nguyen, Quynh-Tho Thi Nguyen, Thai-Hoa Thi Nguyen, Chu Van Nguyen, Ha Thu Le, Mai-Lan Thi Nguyen, Trieu Vu Nguyen, Vu Uyen Tran, Bac An Luong, Le Gia Hoang Le, Quoc Chuong Ho, Hong-Anh Thi Pham, Binh Thanh Vo, Luan Thanh Nguyen, Anh-Thu Huynh Dang, Sinh Duy Nguyen, Duc Minh Do, Thanh-Thuy Thi Do, Anh Vu Hoang, Kiet Truong Dinh, Minh-Duy Phan, Hoa Giang, Le Son Tran

Supplementary figure legends

Figure S1: The mutation profiles of 51 Vietnamese NSCLC patients who developed acquired resistance to first and second generation EGFR TKI
(A) Computation plot of all mutational profiles in 51 Vietnamese NSCLC patients. Each vertical line of blocks represents a patient. Mutations with known resistant, sensitive, predicted sensitive activity or unknown activity to EGFR TKI drugs were colored in red, blue, green and pink, respectively.

Figure S2: Correlation between the abundance of on-target resistance mutations including (A) *EGFR* amplification (n=8) and (B) T790M (n=33) with their corresponding sensitizing VAFs as expressed by linear regression and Pearson's correlation.

Figure S3: EGFR on-target resistance mechanisms showed genome-wide hypo-methylation and CNA changes distinct from EGFR off-target resistance mechanisms
(A) Patients' ages of on-target mutation cases (T790M, *EGFR* amplification and co-occurring T790M and *EGFR* amplification, n=11) and off-target mutation (HER2 and MET amplification, n=7). Data are shown as median, ns: not significant (Mann-Whitney test).
Quantification of hypo-methylated bins (B) and CNA bins (C) in each autosome. Individual values are overlaid on top of box-plots.

Figure S4: Correlations between hypo-methylation, CNA changes and T790M VAF and the duration response to TKI treatment
Linear regression and Pearson's correlation between proportion of CNA bins (A), hypo-methylation bins (B), T790M VAF (C) and time to treatment resistance (TTTR) in 5 cases with T790M.

Figure S5: Graphical summary of the changes observed in different acquired resistance mutation profiles. The EGFR on-target group (T790M and *EGFR* amplification) acquired extensive global methylation, genome instability and hyper-methylation at transcriptional regulation regions of HOX genes. In contrast, fewer changes in DNA methylation and CNA were observed in the EGFR off-target group

(HER2 and MET amplification) during the response to EGFR 1G and 2G TKI treatment.
