# Supplementary material for: Liquid biopsy uncovers distinct patterns of DNA methylation and copy number changes in NSCLC patients with different EGFR-TKI resistant mutations
Source: Sci Rep. 2021 Aug 12;11:16436. doi: 10.1038/s41598-021-95985-6 (PMC8361064; doi:10.1038/s41598-021-95985-6)
Supplement: Supplementary file 2 — Supplementary Figure S1. [file 41598_2021_95985_MOESM2_ESM.pdf]

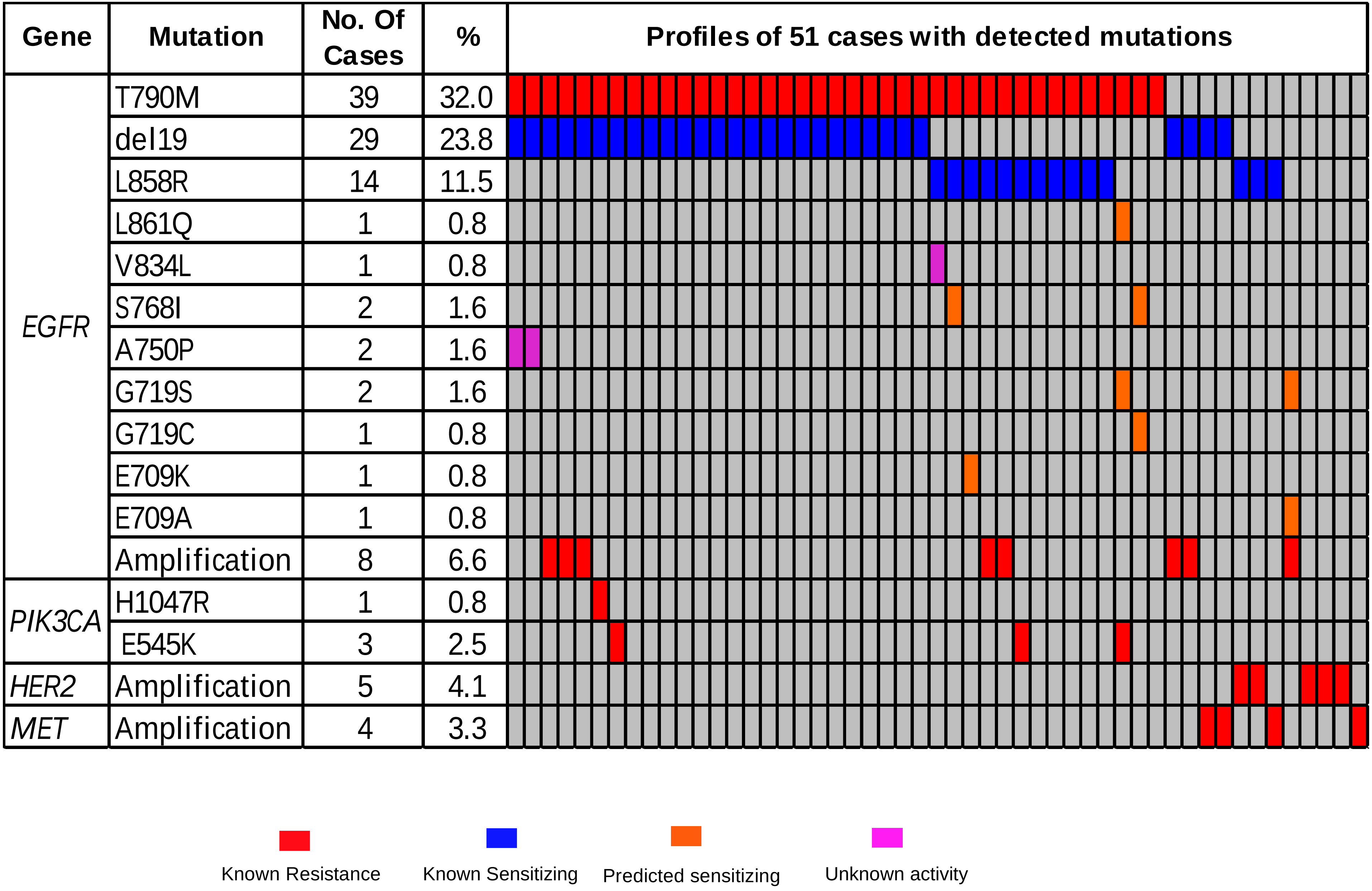

Gene	Mutation	No. Of Cases	%	Profiles of 51 cases with detected mutations
EGFR	T790M	39	32.0	
	del19	29	23.8	
	L858R	14	11.5	
	L861Q	1	0.8	
	V834L	1	0.8	
	S768I	2	1.6	
	A750P	2	1.6	
	G719S	2	1.6	
	G719C	1	0.8	
	E709K	1	0.8	
	E709A	1	0.8	
	Amplification	8	6.6	
PIK3CA	H1047R	1	0.8	
	E545K	3	2.5	
HER2	Amplification	5	4.1	
MET	Amplification	4	3.3	
