# Supplementary figures and images for: Liquid biopsy uncovers distinct patterns of DNA methylation and copy number changes in NSCLC patients with different EGFR-TKI resistant mutations
Source: Sci Rep. 2021 Aug 12;11:16436. doi: 10.1038/s41598-021-95985-6 (PMC8361064; doi:10.1038/s41598-021-95985-6)

A

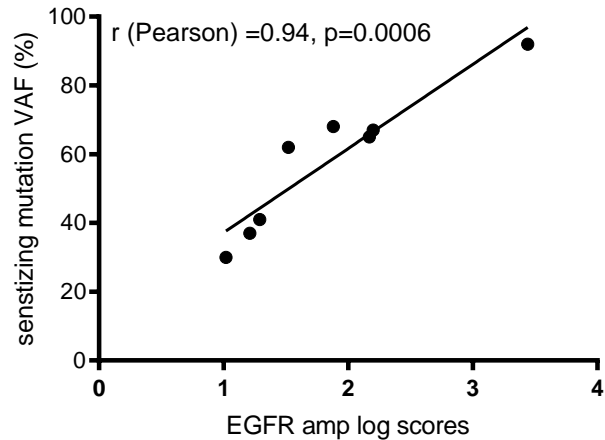

B

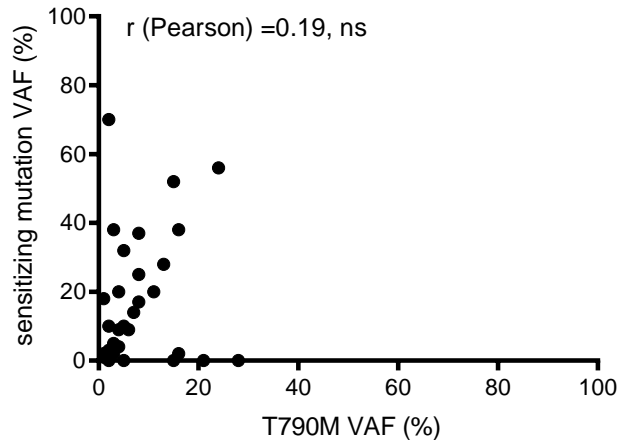

Supplement: Supplementary file 3 — Supplementary Figure S2. [file 41598_2021_95985_MOESM3_ESM.pdf]

**A**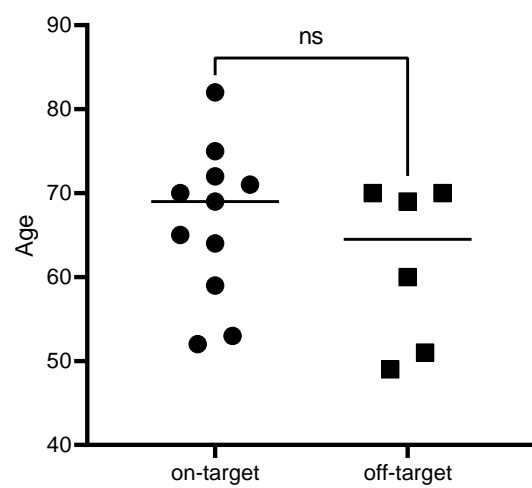**B**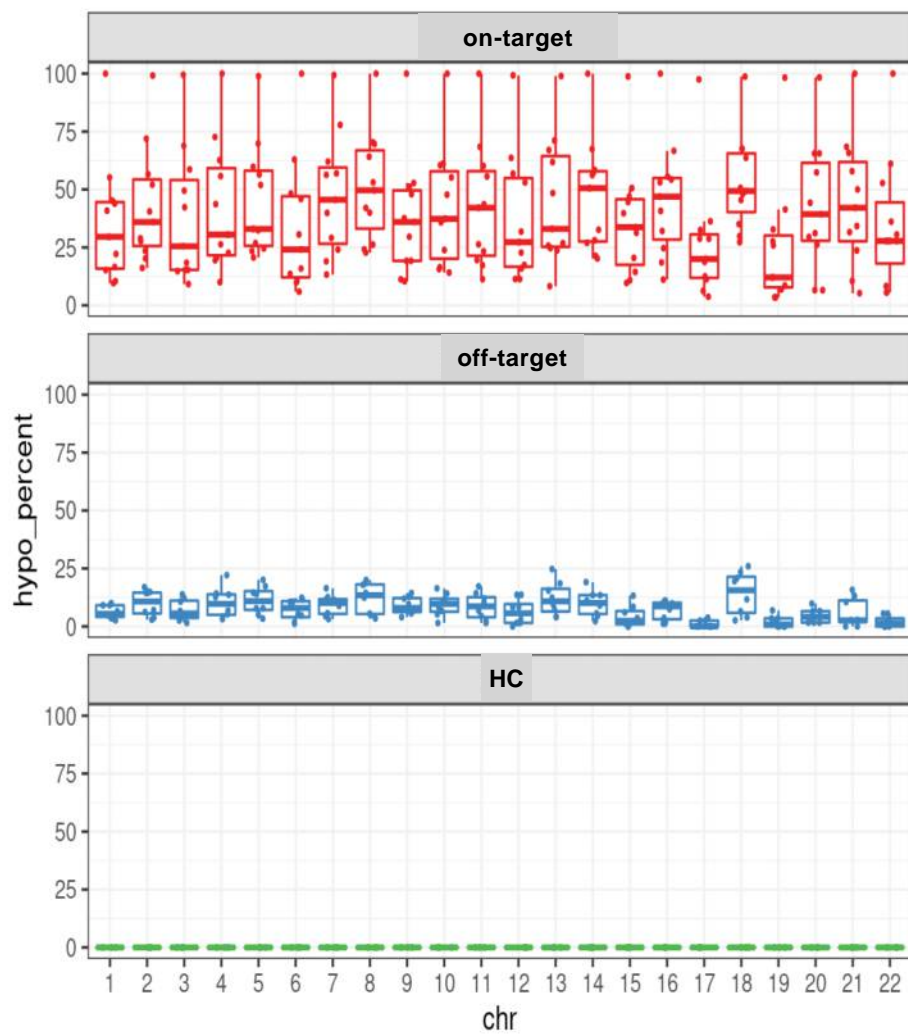**C**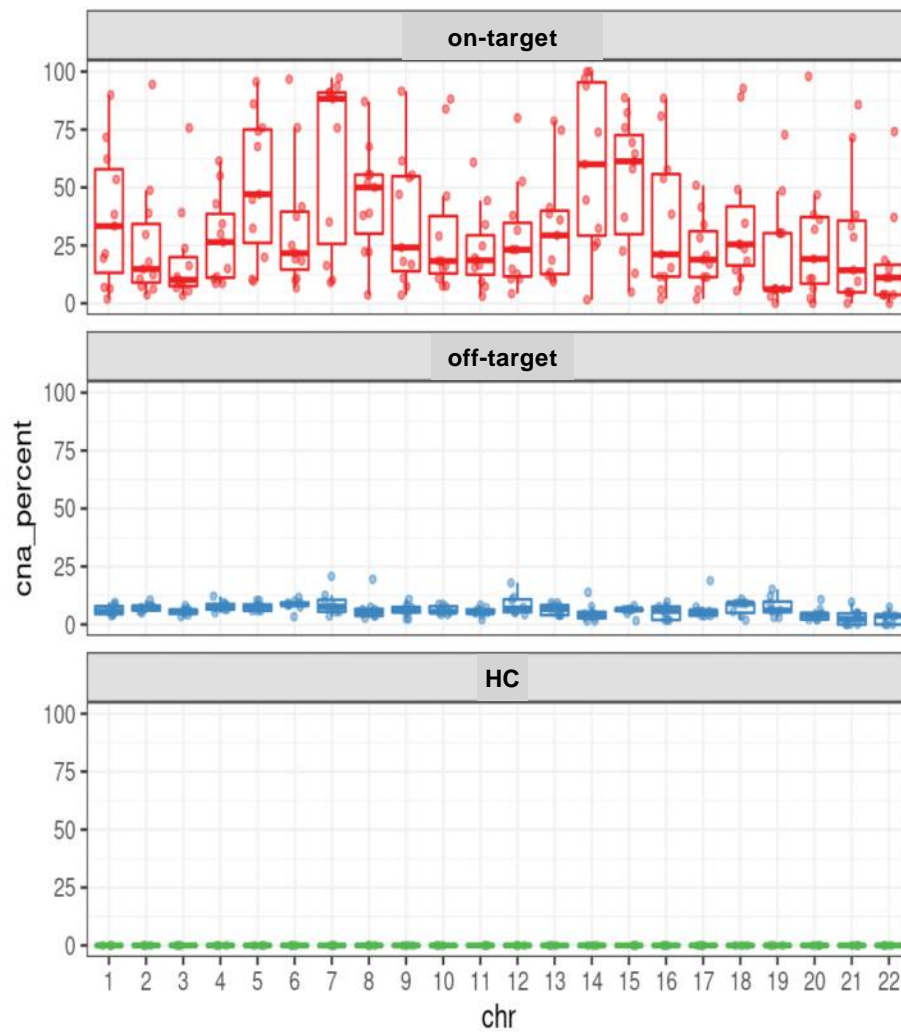

Supplement: Supplementary file 4 — Supplementary Figure S3. [file 41598_2021_95985_MOESM4_ESM.pdf]

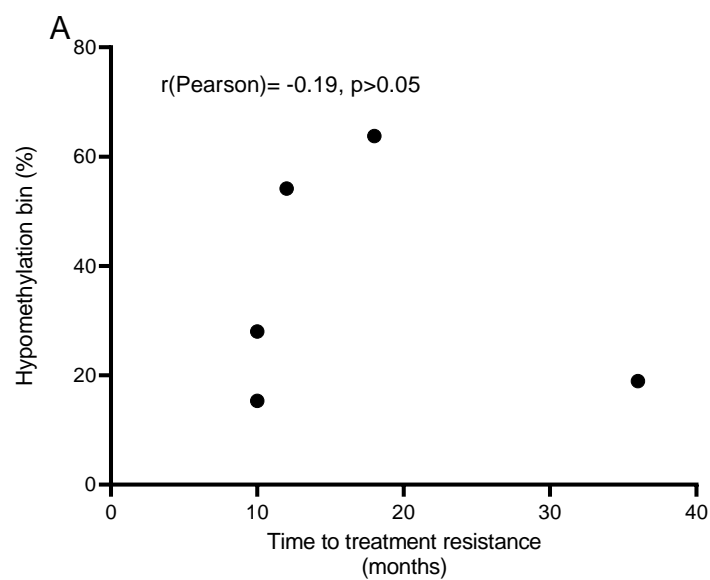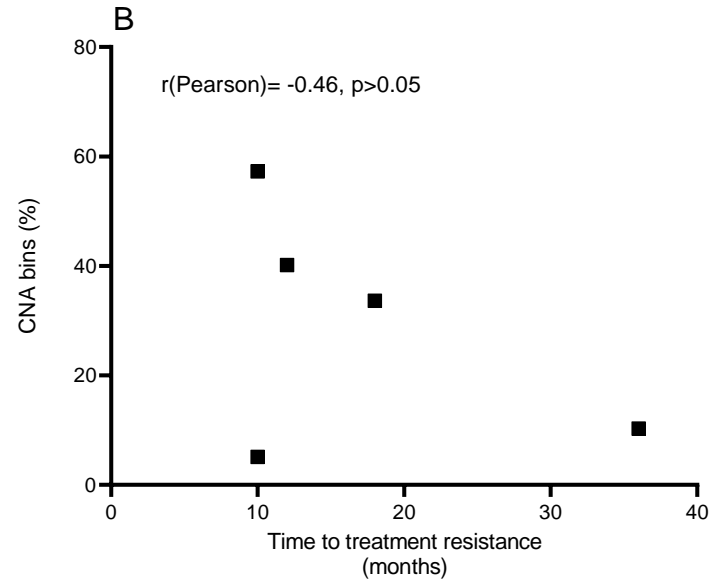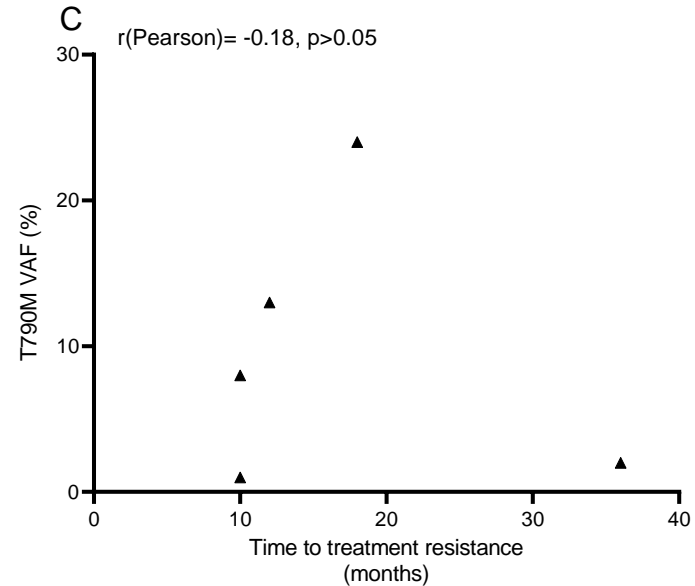

Supplement: Supplementary file 5 — Supplementary Figure S4. [file 41598_2021_95985_MOESM5_ESM.pdf]

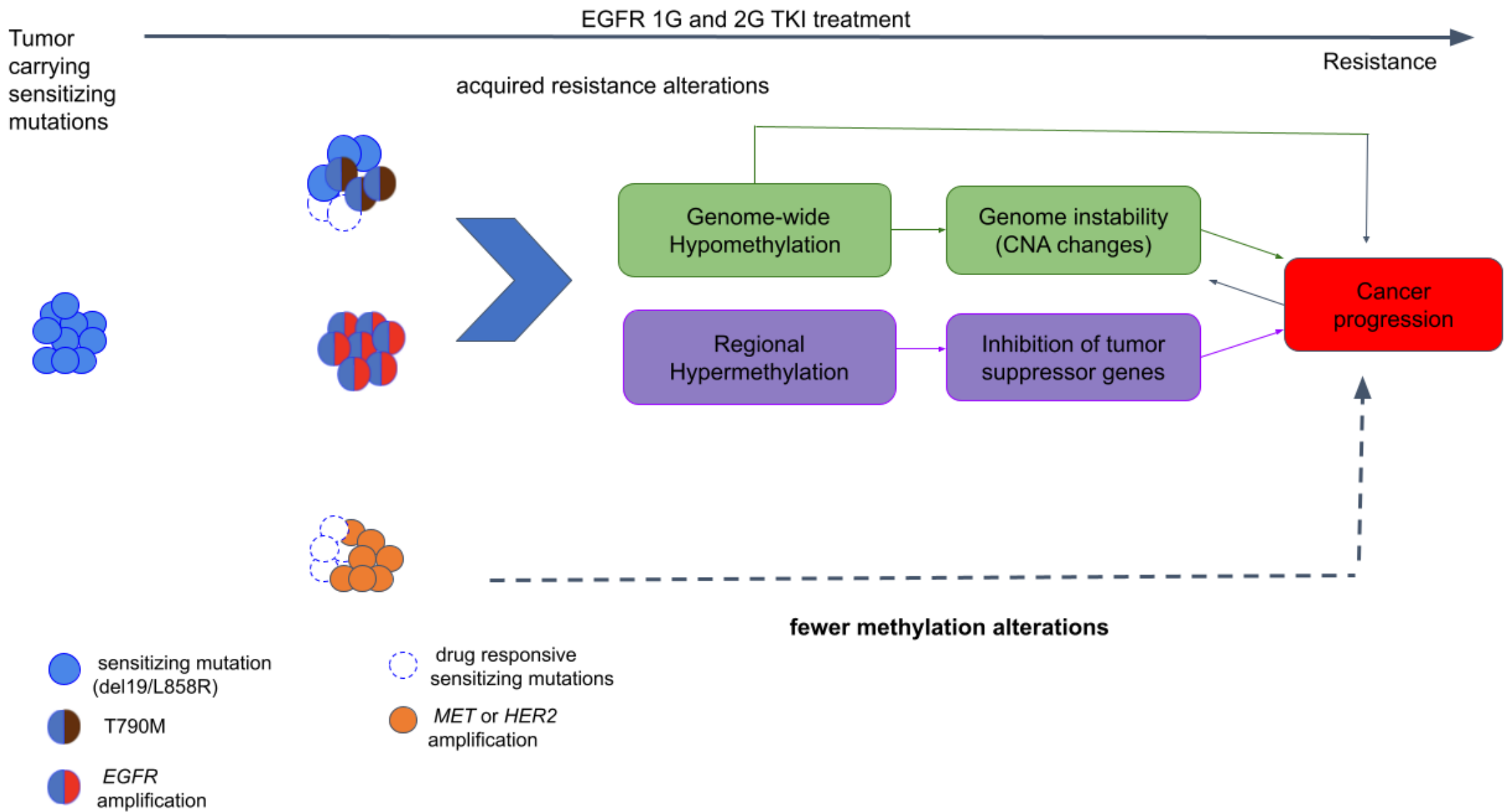

Supplement: Supplementary file 6 — Supplementary Figure S5. [file 41598_2021_95985_MOESM6_ESM.pdf]
