# Supplementary material for: Liquid biopsy uncovers distinct patterns of DNA methylation and copy number changes in NSCLC patients with different EGFR-TKI resistant mutations
Source: Sci Rep. 2021 Aug 12;11:16436. doi: 10.1038/s41598-021-95985-6 (PMC8361064; doi:10.1038/s41598-021-95985-6)
Supplement: Supplementary file 7 — Supplementary Table S1. [file 41598_2021_95985_MOESM7_ESM.pdf]

Liquid biopsy uncovers distinct patterns of DNA methylation and copy number changes in NSCLC patients with different EGFR-TKI resistant mutations

Hoai-Nghia Nguyen, Ngoc-Phuong Thi Cao, Thien-Chi Van Nguyen, Khang Nguyen Duy Le, Dat Thanh Nguyen, Quynh-Tho Thi Nguyen, Thai-Hoa Thi Nguyen, Chu Van Nguyen, Ha Thu Le, Mai-Lan Thi Nguyen, Trieu Vu Nguyen, Vu Uyen Tran, Bac An Luong, Le Gia Hoang Le, Quoc Chuong Ho, Hong-Anh Thi Pham, Binh Thanh Vo, Luan Thanh Nguyen, Anh-Thu Huynh Dang, Sinh Duy Nguyen, Duc Minh Do, Thanh-Thuy Thi Do, Anh Vu Hoang, Kiet Truong Dinh, Minh-Duy Phan, Hoa Giang, Le Son Tran

Table S1 Clinical characteristics and mutation results of 122 Vietnamese NSCLC patients with resistance to TKI drugs
AC: Adenocarcinoma; SCC: squamous cell carcinoma; VAF: variant allelic frequency; amp: amplification

Case No.	SampleID	Age	Gender	Pathology	Stage	Smoking	First line treatment	Second line treatment	Time to Treatment Discontinuation (months)	Sensitizing mutations		Rare *EGFR* mutations		EGFR T790M		Mutations in bypass pathways	
										Mutation	VAF	Mutation	VAF	Mutation	VAF		
1	L10005	53	Male	AC	IV	NA	TKI I/II			L858R	4						
2	L10007	50	Female	AC	IV	NA	TKI I/II		7	Del19	2			T790M	16		
3	L10024	69	Female	SCC	IV	No	TKI I/II										
4	L10046	75	Female	AC	IV	No	TKI I/II		18	L858R	56			T790M	24		
5	L10113	59	Female	AC	NA	NA	TKI I/II										
6	L10188	44	Female	NA	NA	NA	Erlotinib		24								
7	L10190	49	Male	AC	IV	No	TKI I/II		24	Del19	45						
8	L10225	67	Female	AC	IV	NA	Erlotinib										
9	L10250	79	Female	AC	IV	No	Erlotinib		24								
10	L10256	34	NA	AC	IV	NA	Erlotinib			L858R	20	E709K	20	T790M	11		
11	L10258	76	Female	AC	IV	NA	Erlotinib			Del19	20			T790M	4	PIK3CA H1047R	23
12	L10261	73	Female	AC	IV	NA	Erlotinib										
13	L10282	66	Male	AC	IV	NA	Erlotinib			L861Q	70	G719S	67	T790M	2	PIK3CA E545K	4
14	L10295	52	Female	AC	IV	NA	Gefitinib										
15	L10372	58	Female	AC	IV	NA	Erlotinib		14								
16	L10381	64	Male	AC	IV	NA	Erlotinib		13	Del19	30			T790M	16	EGFR amp	
17	L10382	52	Female	SCC	IV	NA	Erlotinib		10	Del19	2			T790M	1		
18	L10383	53	Female	AC	IV	No	Erlotinib			Del19	1 read						
19	L10435	57	Female	AC	IV	NA	TKI I/II		24								
20	L10454	49	Female	NA	IV	No	Erlotinib			del19	1						
21	L10460	72	Male	AC	IV	NA	Erlotinib			L858R	1 read			T790M	1 read		
22	L10466	49	Female	AC	IV	NA	Erlotinib										
23	L10509	53	NA	NA	IV	NA	Erlotinib			del19	2						
24	L10533	76	Female	AC	IV	NA	Erlotinib		15	L858R	4	V834L	4	T790M	3		
25	L10540	68	Female	SCC	IV	NA	Erlotinib		7	del19	18			T790M	1	PIK3CA E545K	26
26	L10581	66	Male	AC	IV	NA	Erlotinib		15			S768<br>G719CI	26<br>23	T790M	15		
27	L10582	47	Male	AC	IV	NA	Erlotinib		8			L858R	2				
28	L10606	63	Female	AC	NA	NA	TKI I/II		24	del19	1	A750P	1	T790M	1		
29	L10611	62	Male	AC	IV	NA	Afatinib		7								
30	L10672	59	Female	NA	NA	NA	Erlotinib	Afatinib	36	del19	92					EGFR amp	
31	L10682	73	Female	AC	IV	NA	Erlotinib		48			del19	1				
32	L10694	53	Female	NA	III	No	Gefitinib		22								
33	L10695	70	Female	NA	NA	NA	Gefitinib		14	L858R	68			T790M	36	EGFR amp	
34	L10710	54	Female	AC	IV	NA	Erlotinib					del19	1				
35	L10719	50	Male	NA	IV	NA	Erlotinib										
36	L10741	73	Male	NA	IV	Yes	Erlotinib										
37	L10743	63	Female	AC	IV	No	Erlotinib					L858R	6				
38	L10749	69	Female	AC	IV	NA	Erlotinib		16								
39	L10750	59	Female	AC	IV	NA	TKI I/II		12								
40	L10771	68	Female	AC	IV	NA	Gefitinib					del19	53	T790M	28		
41	L10777	83	Male	SCC	IV	NA	Erlotinib		14			G719C<br>S768I	16<br>21	T790M	21		
42	L10806	60	Female	AC	IV	NA	Gefitinib										
43	L10808	68	Female	AC	IV	NA	Gefitinib					del19	12	T790M	5		
44	L10812	39	Male	NA	IV	NA	Erlotinib		7			del19	5	T790M	2		
45	L10814	69	Male	NA	IV	Yes	Gefitinib		12	del19	62	G719S<br>E709A	62<br>62			EGFR amp	
46	L10817	58	Female	AC	IV	NA	Erlotinib		5	del19	1						
47	L10821	65	Male	NA	NA	No	Gefitinib		8	del19	37					EGFR amp	
48	L10842	52	Male	AC	NA	Yes	Erlotinib		14	del19	10			T790M	2		
49	L10855	69	Female	AC	IV	NA	Gefitinib		12	del19	2						
50	L10857	71	Female	NA	IV	NA	Erlotinib		6								
51	L10860	51	Male	NA	IV	No	Gefitinib			del19	3			T790M	2		
52	L10882	53	Female	AC	IV	NA	Erlotinib		15	del19	65			T790M	13	EGFR amp	
53	L10897	57	Female	NA	NA	No	Erlotinib		14	L858R	17			T790M	8		
54	L10900	82	Female	NA	NA	NA	Gefitinib		36	del19	2			T790M	2		
55	L10902	61	Male	AC	IV	No	Erlotinib		12								
56	L10921	67	Male	AC	IV	No	Erlotinib										
57	L10953	41	Female	AC	IV	NA	Erlotinib		>3	del19	9			T790M	6		
58	L10955	71	Female	AC	IV	NA	Erlotinib		12	del19	28			T790M	13		
59	L10956	61	Male	NA	IV	Yes	Gefitinib										
60	L10994	48	Male	AC	NA	No	Erlotinib		6	del19	20			T790M	4		
61	L11018	67	Female	AC	IV	NA	Gefitinib		>6	del19	38			T790M	16		
62	L11022	72	Female	AC	IV	NA	Erlotinib		20								
63	L11027	56	Male	AC	IV	NA	Erlotinib										
64	L11032	57	Female	NA	IV	NA	Gefitinib										
65	L11034	72	Female	AC	IV	NA	Erlotinib		10	L858R	37			T790M	8		
66	L11044	64	Female	AC	IV	NA	Erlotinib		18								
67	L11094	62	Male	NA	IV	NA	Erlotinib			L858R	41			T790M	15	EGFR amp	
68	L11118	44	Male	NA	IV	NA	Erlotinib	Afatinib		del19	67			T790M	70	EGFR amp	
69	L11119	68	Male	AC	IV	NA	Erlotinib			del19	52			T790M	15		
70	L11133	70	Female	AC	IV	No	Erlotinib									HER2 amp	
71	L11135	NA	Female	AC	NA	NA	Erlotinib			del19	2			T790M	3		
72	L11190	52	Male	NA	NA	NA	TKI I/II			del19	50						
73	L11196	60	Female	AC	IV	No	Erlotinib			L858R	10						
74	L11228	57	Female	NA	IV	No	Erlotinib			L858R	32			T790M	5	PKI3CA E545K	2
75	L11258	54	Male	AC	IV	Yes	Erlotinib			del19	4			T790M	4		
76	L11266	65	Female	AC	NA	NA	TKI I/II			L861Q	19						

77	L11281	59	Female	NA	NA	No	afatinib		12							HER2 amp	
78	NA001	55	Female	AC	IV	No	TKI I/II		32	L858R	44						
79	NA002	29	Female	AC	IV	No	TKI I/II			del19	72						
80	NA004	66	Female	AC	IV	No	TKI I/II		>18	Del19	33						
81	NA005	67	Male	AC	IV	Yes	TKI I/II			Del19	1						
82	NA007	58	Female	NA	IV	No	TKI I/II										
83	NA008	60	Male	NA	NA	NA	Erlotinib			Del19	2						
84	NA009	61	Male	NA	IV	NA	Erlotinib			Del19	1						
85	NA010	58	Male	NA	NA	NA	Erlotinib			Del19	9			T790M	4		
86	NA011	NA	Female	NA	NA	NA	Erlotinib									MET amp	
87	NA012	49	Male	NA	NA	NA	Erlotinib			Del19	1					MET amp	
88	NA014	50	Male	NA	IV	NA	Erlotinib			del19	38	A750P	43	T790M	3		
89	NA015	45	Male	AC	IV	Yes	TKI I/II		21								
90	NA016	51	Female	AC	IV	No	TKI I/II		21	Del19	1					MET amp	
91	NA018	60	Female	AC	IV	NA	Erlotinib										
92	NA019	50	Male	AC	IV	NA	Erlotinib			L858R	20						
93	NA020	63	Female	NA	NA	NA	Erlotinib										
94	NA021	63	Female	AC	IV	No	Erlotinib		29	Del19	19						
95	NA022	69	Male	AC	IV	No	Erlotinib	Gefitinib	9								
96	NA023	52	Male	AC	IV	Yes	Erlotinib	Gefitinib	11								
97	NA024	55	Male	AC	IV	Yes	Erlotinib		11	L858R	1						
98	NA025	50	Male	AC	IV	NA	Erlotinib										
99	NA026	67	Male	AC	IV	Yes	Erlotinib										
100	NA027	75	Female	AC	IV	No	Erlotinib		24	L858R	12					HER2 amp	
101	NA028	64	Female	AC	IV	No	Erlotinib			L858R	14						
102	NA030	58	Male	AC	IV	NA	Afatinib										
103	NA031	66	Female	AC	IV	NA	Erlotinib			L858R	6	E709K	4				
104	NA032	77	Female	AC	IV	NA	Erlotinib			L858R	3						
105	NA033	63	Female	AC	IV	No	Erlotinib		6								
106	NA034	64	Female	AC	IV	No	Erlotinib		26								
107	NA035	70	Male	AC	IV	Yes	Erlotinib	Afatinib	2	L858R	5					HER2 amp	
108	NA036	73	Male	AC	IIIB	NA	Erlotinib		24	del19	6						
109	NA038	49	Female	AC	IV	NA	Erlotinib			L858R	7						
110	NA039	64	Female	AC	IV	No	Erlotinib										
111	NA040	65	Male	AC	IV	NA	Erlotinib		24	Del19	7						
112	NA041	74	Female	AC	IV	No	Erlotinib		21	L858R	10			T790M	5		
113	NA042	58	Female	AC	IV	No	Erlotinib		8								
114	NA043	64	Male	AC	IV	Yes	Erlotinib										
115	NA044	60	Male	AC	IV	Yes	Erlotinib			L858R	2					MET amp	
116	NA045	69	Female	NA	NA	NA	Erlotinib									HER2 amp	
117	NA046	39	Female	AC	IV	NA	Erlotinib		8								
118	NA047	60	Male	AC	IV	NA	Erlotinib		18	del19	25			T790M	8		
119	NA048	47	Male	AC	IV	Yes	Erlotinib		6								
120	NA049	70	Male	NA	NA	NA	Erlotinib			L858R	5	S768I	6	T790M	3		
121	NA050	64	Female	AC	IV	No	Erlotinib		>24	Del19	14			T790M	7		
122	NA051	57	Male	NA	NA	Yes	Erlotinib										
