# Supplementary material for: Liquid biopsy uncovers distinct patterns of DNA methylation and copy number changes in NSCLC patients with different EGFR-TKI resistant mutations
Source: Sci Rep. 2021 Aug 12;11:16436. doi: 10.1038/s41598-021-95985-6 (PMC8361064; doi:10.1038/s41598-021-95985-6)
Supplement: Supplementary file 8 — Supplementary Table S2. [file 41598_2021_95985_MOESM8_ESM.pdf]

Liquid biopsy uncovers distinct patterns of DNA methylation and copy number changes in NSCLC patients with different EGFR-TKI resistant mutations

Hoai-Nghia Nguyen, Ngoc-Phuong Thi Cao, Thien-Chi Van Nguyen, Khang Nguyen Duy Le, Dat Thanh Nguyen, Quynh-Tho Thi Nguyen, Thai-Hoa Thi Nguyen, Chu Van Nguyen, Ha Thu Le, Mai-Lan Thi Nguyen, Trieu Vu Nguyen, Vu Uyen Tran, Bac An Luong, Le Gia Hoang Le, Quoc Chuong Ho, Hong-Anh Thi Pham, Binh Thanh Vo, Luan Thanh Nguyen, Anh-Thu Huynh Dang, Sinh Duy Nguyen, Duc Minh Do, Thanh-Thuy Thi Do, Anh Vu Hoang, Kiet Truong Dinh, Minh-Duy Phan, Hoa Giang, Le Son Tran

Table S2: 450 target regions

ID	chrom	start	end	gene	strand
1	chr1	1098979	1099212	C1orf159_TTLL10	+
2	chr1	2375304	2375684	PEX10_PLCH2	+
3	chr1	2705890	2706174	ACTRT2_MMEL1	+
4	chr1	2706288	2706691	ACTRT2_MMEL1	+
5	chr1	6125059	6125309	KCNAB2_CHD5	+
6	chr1	7728778	7728879	VAMP3_CAMTA1	+
7	chr1	7729026	7729031	VAMP3_CAMTA1	+
8	chr1	9377932	9378290	SLC25A33_SPSB1	+
9	chr1	15672438	15672742	EFHD2_TMEM51	+
10	chr1	29586187	29586646	PTPRU	+
11	chr1	37941178	37941397	ZC3H12A_MEAF6	+
12	chr1	44015716	44015926	KDM4A_PTPRF	+
13	chr1	48173969	48174156	FOXD2_TRABD2B	+
14	chr1	50881990	50882155	DMRTA2_ELAVL4	+
15	chr1	50884549	50885150	DMRTA2_ELAVL4	+
16	chr1	50885338	50885470	DMRTA2_ELAVL4	+
17	chr1	63785840	63786084	FOXD3	+
18	chr1	68025114	68025305	SERBP1_GADD45A	+
19	chr1	91183443	91183587	BARHL2	-
20	chr1	110334811	110334876	CSF1_EPS8L3	+
21	chr1	110334989	110335019	CSF1_EPS8L3	+
22	chr1	110612460	110612814	ALX3	+
23	chr1	110626174	110626397	UBL4B_ALX3	+
24	chr1	111813487	111813699	CHIA_CHI3L2	+
25	chr1	111813838	111814003	CHIA_CHI3L2	+
26	chr1	119522367	119522549	SPAG17_TBX15	+
27	chr1	119526985	119527255	SPAG17_TBX15	+
28	chr1	119532693	119532983	TBX15	-
29	chr1	119535682	119535909	TBX15	-
30	chr1	145075754	145075942	ENSG00000255168_PDE4DIP	+
31	chr1	145562790	145563083	PIAS3_ITGA10	+
32	chr1	155265002	155265487	PKLR_HCN3	+
33	chr1	156092387	156092640	SEMA4A_LMNA	+
34	chr1	156406407	156406632	C1orf61_MEF2D	+
35	chr1	156611841	156612098	BCAN	+
36	chr1	161038973	161039034	ARHGAP30	+
37	chr1	166890392	166890611	TADA1_ILDR2	+
38	chr1	221050087	221050260	HLX	+
39	chr1	221050372	221050608	HLX	+
40	chr1	237205530	237205730	RYR2	+
41	chr1	237205909	237206236	RYR2	+
42	chr1	240161147	240161311	FMN2_CHRM3	+
43	chr1	240161424	240161559	FMN2_CHRM3	+
44	chr1	248020558	248020995	TRIM58	+
45	chr10	555353	555459	DIP2C_ZMYND11	+
46	chr10	3514754	3514827	PITRM1_KLF6	+
47	chr10	7449724	7449927	PRKCQ_SFMBT2	+
48	chr10	7455102	7455242	SFMBT2	-
49	chr10	8094211	8094348	GATA3	+
50	chr10	23480598	23480841	PTF1A	+
51	chr10	28034310	28034506	MKX	-
52	chr10	28034644	28034857	MKX	-
53	chr10	72320312	72320623	PRF1_PALD1	+
54	chr10	74078123	74078156	DNAJB12_DDIT4	+
55	chr10	74210234	74210297	DNAJB12_MICU1	+
56	chr10	94451964	94452121	EXOC6_HHEX	+
57	chr10	94822336	94822766	CYP26A1_CYP26C1	+
58	chr10	94828102	94828377	CYP26A1_CYP26C1	+
59	chr10	99080841	99081075	FRAT1_FRAT2	+
60	chr10	102899822	102900142	TLX1_LBX1	+
61	chr10	103044094	103044401	BTRC_LBX1	+
62	chr10	108923780	108924304	SORCS1	+
63	chr10	110672156	110672326	NONE	+

64	chr10	123923130	123923372	PLEKHA1_TACC2	+
65	chr10	123923732	123924175	PLEKHA1_TACC2	+
66	chr10	124905496	124906129	HMX2	+
67	chr10	124910752	124910996	BUB3	+
68	chr10	126135970	126136163	OAT_NKX1-2	+
69	chr10	129534706	129534913	FOXI2	+
70	chr10	130732305	130732507	MKI67_MGMT	+
71	chr10	131213445	131213769	MGMT	+
72	chr10	131223411	131223630	MGMT	+
73	chr10	17271288	17271504	VIM	-
74	chr10	131265516	131265596	MGMT	+
75	chr10	93392882	93392981	PPP1R3C	-
76	chr10	88683960	88684081	BMPR1A	+
77	chr10	17495750	17495871	ST8SIA6	+
78	chr10	17496615	17496781	ST8SIA6	-
79	chr11	280945	281044	ATHL1_NLRP6	+
80	chr11	281278	281496	ATHL1_NLRP6	+
81	chr11	636846	637203	DRD4	+
82	chr11	1331724	1331858	TOLLIP	-
83	chr11	1331978	1332017	TOLLIP	-
84	chr11	1683157	1683261	KRTAP5-6_KRTAP5-5	+
85	chr11	31837358	31837807	PAX6_ELP4	-
86	chr11	58672851	58673049	GLYATL2_GLYATL1	+
87	chr11	61536887	61537195	MYRF_TMEM258	+
88	chr11	62211803	62212115	SCGB1A1_AHNAK	+
89	chr11	63687127	63687366	RCOR2	-
90	chr11	76033076	76033392	WNT11_PRKRIR	+
91	chr11	76750654	76750944	CAPN5_B3GNT6	+
92	chr11	94600602	94600758	AMOTL1_CWC15	+
93	chr11	125036382	125036637	PKNOX2_FEZ1	-
94	chr11	30607008	30607129	MPPED2Ê	-
95	chr11	131780423	131780494	OPCML	+
96	chr12	6184401	6184680	ANO2_VWF	+
97	chr12	6664346	6664537	IFFO1	-
98	chr12	6664804	6664910	IFFO1	-
99	chr12	6665056	6665530	IFFO1	-
100	chr12	7072502	7072697	PHB2_PTPN6	+
101	chr12	25055818	25056391	BCAT1	-
102	chr12	33592318	33592537	SYT10	-
103	chr12	33592773	33592954	SYT10	-
104	chr12	54441275	54441495	HOXC4_HOXC5	+
105	chr12	58013494	58013742	SLC26A10	+
106	chr12	63544447	63544882	AVPR1A	+
107	chr12	66275927	66276102	HMGA2_ENSG00000228144	-
108	chr12	70051532	70051714	LRRC10_BEST3	+
109	chr12	81312904	81312966	LIN7A_MYF5	+
110	chr12	104193771	104193807	NT5DC3_STAB2	+
111	chr12	104193911	104193975	NT5DC3_STAB2	+
112	chr12	113901278	113901427	LHX5_SDSL	+
113	chr12	113901942	113901976	LHX5_SDSL	+
114	chr12	113902080	113902304	LHX5_SDSL	+
115	chr12	113917404	113917703	LHX5_RBM19	+
116	chr12	114840799	114841333	RBM19_TBX5	+
117	chr12	114886354	114886619	TBX5_TBX3	+
118	chr12	122473180	122473464	MLXIP_BCL7A	+
119	chr12	124941685	124942045	NCOR2_ZNF664	+
120	chr12	132258450	132258649	MMP17_SFSWAP	+
121	chr12	133481286	133481642	CHFR_ZNF605	+
122	chr12	25101992	25102093	BCAT1	+
123	chr12	65515256	65515458	WIF1	-
124	chr13	21520218	21520413	XPO4_LATS2	+
125	chr13	25320074	25320295	RNF17_ATP12A	+
126	chr13	25320419	25320698	RNF17_ATP12A	+
127	chr13	51417487	51417687	RNASEH2B_DLEU1	+
128	chr13	53313409	53313622	LECT1	+

129	chr13	100608122	100608388	ZIC5_CLYBL	+
130	chr13	100649566	100649848	PCCA_ZIC2	+
131	chr13	109147997	109148189	MYO16_TNFSF13B	+
132	chr13	109148317	109148468	MYO16_TNFSF13B	+
133	chr13	111178258	111178399	RAB20_COL4A2	+
134	chr13	112547627	112548039	SOX1_TEX29	+
135	chr13	112717041	112717439	SOX1	+
136	chr13	113436467	113436692	MCF2L_ATP11A	+
137	chr13	28543014	28543097	CDX2	+
138	chr13	36920592	36920685	SPG20	+
139	chr14	21093698	21093907	RNASE12_OR6S1	+
140	chr14	55243068	55243425	GCH1_SAMD4A	+
141	chr14	56705455	56705503	TMEM260_PELI2	+
142	chr14	57264957	57265561	OTX2_TMEM260	-
143	chr14	57275767	57276040	OTX2_TMEM260	-
144	chr14	60207779	60207902	RTN1_JKAMP	+
145	chr14	77491953	77492311	IRF2BPL_VASH1	+
146	chr14	93153542	93153863	LGMN_RIN3	+
147	chr14	93154069	93154333	LGMN_RIN3	+
148	chr14	93522385	93522387	ITPK1_CHGA	+
149	chr14	97499574	97500077	VRK1	+
150	chr14	100857048	100857281	WARS_BEGAIN	+
151	chr14	105750446	105750677	PACS2_BTBD6	+
152	chr14	106330433	106330539	TMEM121	+
153	chr14	94255240	94255339	PRIMA1	-
154	chr15	22908513	22908658	CYFIP1_NIPA2	+
155	chr15	29395480	29395729	NDNL2_APBA2	+
156	chr15	41787837	41788308	ITPKA_LTK	+
157	chr15	41793266	41793500	ITPKA_LTK	+
158	chr15	41795000	41795251	ITPKA_LTK	+
159	chr15	45427308	45427482	DUOX1_SHF	+
160	chr15	53083366	53083605	ONECUT1	-
161	chr15	68114384	68114554	PIAS1_SKOR1	+
162	chr15	76639691	76639823	ISL2_SCAPER	+
163	chr16	11170223	11170403	SOCS1_CIITA	+
164	chr16	11327008	11327232	SOCS1_CIITA	+
165	chr16	22825609	22826202	HS3ST2	+
166	chr16	27240309	27240515	KDM8_NSMCE1	+
167	chr16	51189915	51190260	SALL1	-
168	chr16	57654201	57654538	GPR56_GPR114	+
169	chr16	70771557	70771814	MTSS1L_VAC14	+
170	chr16	86321477	86321818	FOXF1_IRF8	+
171	chr16	86612367	86612598	FOXL1	+
172	chr16	86612922	86613063	FOXL1	+
173	chr16	86613195	86613349	FOXL1_FBXO31	+
174	chr16	88769853	88770135	CTU2_RNF166	+
175	chr16	58497395	58497458	NDRG4	+
176	chr17	25867546	25867639	LGALS9_KSR1	+
177	chr17	25867745	25867753	LGALS9_KSR1	+
178	chr17	35165557	35165914	LHX1_MRM1	+
179	chr17	35299775	35299991	AATF_LHX1	+
180	chr17	37321625	37321818	PLXDC1_ARL5C	+
181	chr17	46621708	46621880	HOXB1_HOXB2	+
182	chr17	46622098	46622227	HOXB1_HOXB2	+
183	chr17	46832289	46832580	HOXB13_TTLL6	+
184	chr17	48546637	48546885	CHAD	-
185	chr17	55520553	55520788	MSI2_ENSG00000166329	+
186	chr17	56405881	56406036	BZRAP1	+
187	chr17	59482165	59482348	TBX4_TBX2	+
188	chr17	59529151	59529348	TBX4	+
189	chr17	59532094	59532280	TBX4	+
190	chr17	62775622	62775847	SMURF2_LRRC37A3	+
191	chr17	73483971	73483973	CASKIN2_KIAA0195	+
192	chr17	73484081	73484191	CASKIN2_KIAA0195	+
193	chr17	73636152	73636324	SMIM6_SMIM5	+

194	chr17	73749586	73749805	GALK1_ITGB4	+
195	chr17	75369515	75369860	TNRC6C_SEPT9	+
196	chr17	77386138	77386283	RBFOX3_ENGASE	+
197	chr17	77789574	77789743	CBX8_CBX4	+
198	chr17	78999556	78999742	BAIAP2_CHMP6	+
199	chr17	80846793	80846966	ZNF750_B3GNTL1	+
200	chr17	80847078	80847210	ZNF750_B3GNTL1	+
201	chr18	909017	909219	ADCYAP1	+
202	chr18	47794940	47795094	CCDC11	+
203	chr18	76709408	76709588	SALL3	+
204	chr19	641770	641986	FGF22_RNF126	+
205	chr19	1210276	1210601	STK11_C19orf26	+
206	chr19	1467884	1468076	C19orf25_APC2	+
207	chr19	3688009	3688249	CACTIN_PIP5K1C	+
208	chr19	5229645	5229898	PTPRS_KDM4B	+
209	chr19	11002147	11002208	CARM1_YIPF2	+
210	chr19	11002309	11002379	CARM1_YIPF2	+
211	chr19	15292379	15292632	ILVBL_NOTCH3	+
212	chr19	39754795	39754936	IFNL2	+
213	chr19	42005237	42005494	CEACAM21_ATP5SL	+
214	chr19	46997011	46997223	CCDC8_PPP5D1	+
215	chr19	49238427	49238813	MAMSTR_RASIP1	+
216	chr19	57587879	57587906	ZIM2_USP29	+
217	chr2	468115	468367	FAM150B_TMEM18	+
218	chr2	1036555	1036557	TPO_SNTG2	+
219	chr2	1036660	1036693	TPO_SNTG2	+
220	chr2	2120370	2120628	PXDN_MYT1L	+
221	chr2	5832866	5833085	SOX11	+
222	chr2	10471576	10471983	HPCAL1_ODC1	+
223	chr2	19549979	19550627	NT5C1B-RDH14_OSR1	+
224	chr2	30453574	30453794	LBH	+
225	chr2	31805298	31805547	XDH_MEMO1	+
226	chr2	31805741	31805995	XDH_MEMO1	+
227	chr2	45028955	45029236	SIX3_CAMKMT	+
228	chr2	45227751	45228030	SIX2_SIX3	+
229	chr2	45231696	45231832	SIX2_SIX3	+
230	chr2	47270901	47270918	TTC7A_CALM2	+
231	chr2	47271039	47271132	TTC7A_CALM2	+
232	chr2	66666351	66666865	ETAA1_MEIS1	+
233	chr2	69027038	69027223	ARHGAP25_BMP10	+
234	chr2	73147404	73148014	EMX1_SFXN5	+
235	chr2	74726391	74726810	MRPL53_LBX2	+
236	chr2	85811470	85811855	VAMP5	+
237	chr2	86163782	86164042	ST3GAL5_POLR1A	+
238	chr2	99439492	99439644	MGAT4A_TSGA10	+
239	chr2	105458972	105459192	POU3F3	+
240	chr2	105459316	105459459	POU3F3	+
241	chr2	105460936	105461261	POU3F3	+
242	chr2	106681891	106682238	C2orf40	+
243	chr2	113931517	113931566	PSD4	+
244	chr2	114034363	114034535	PAX8_PSD4	+
245	chr2	131721549	131721891	ARHGEF4_FAM168B	+
246	chr2	131792187	131792403	FAM168B_ARHGEF4	+
247	chr2	162280446	162280694	SLC4A10_TBR1	+
248	chr2	166650478	166650728	GALNT3	+
249	chr2	171679590	171679872	GORASP2_GAD1	+
250	chr2	172965543	172965651	DLX2_DLX1	+
251	chr2	175202258	175202556	SP9_CIR1	+
252	chr2	176947022	176947263	KIAA1715_HOXD10	+
253	chr2	176956534	176956707	HOXD10_HOXD11_	+
254	chr2	176964833	176965036	HOXD10_HOXD11_HOXD12	+
255	chr2	176969341	176969613	HOXD10_HOXD11	+
256	chr2	176987302	176987846	HOXD9	+
257	chr2	176988063	176988243	HOXD8_HOXD9	+
258	chr2	176994727	176995063	HOXD8	+

259	chr2	177017172	177017390	HOXD1_HOXD4	+
260	chr2	177024277	177024416	HOXD1_HOXD4	+
261	chr2	198650908	198651077	BOLL	-
262	chr2	209271306	209271538	PTH2R	+
263	chr2	220313201	220313603	GMPPA_SPEG	+
264	chr2	223163256	223163509	PAX3	-
265	chr2	223163710	223163894	PAX3	-
266	chr2	233925000	233925347	INPP5D	+
267	chr2	242824316	242824578	CXXC11	+
268	chr2	233498328	233498417	EFHD1	+
269	chr20	4803386	4803494	RASSF2A	+
270	chr20	25062234	25062469	VSX1	+
271	chr20	39597821	39598025	MAFB_TOP1	+
272	chr20	48626570	48626729	SNAI1_UBE2V1	+
273	chr20	55202127	55202359	TFAP2C	+
274	chr20	55965076	55965310	RBM38	+
275	chr20	61201084	61201292	GATA5_SLCO4A1	+
276	chr21	38068722	38068902	SIM2	+
277	chr21	38069496	38069867	SIM2	+
278	chr21	38076762	38076953	SIM2_HLCS	+
279	chr21	45604896	45605084	C21orf33_ICOSLG	+
280	chr22	19753101	19753479	TBX1_C22orf29	+
281	chr22	19754706	19754971	TBX1_C22orf29	+
282	chr22	20267802	20267971	RTN4R_DGCR6L	+
283	chr22	20792699	20792898	SCARF2	-
284	chr22	22006593	22006710	PPIL2_SDF2L1	+
285	chr22	26446247	26446382	SEZ6L_MYO18B	+
286	chr22	28195841	28196138	MN1	+
287	chr22	46403725	46404141	PPARA_WNT7B	+
288	chr22	46921637	46921829	CELSR1_TRMU	+
289	chr22	50987269	50987506	TYMP_SYCE3	+
290	chr22	51016357	51016608	CPT1B	-
291	chr3	9178130	9178298	RAD18_SRGAP3	+
292	chr3	52828531	52828707	ITIH3	+
293	chr3	62356119	62356372	FEZF2_PTPRG	+
294	chr3	124860393	124860960	HEG1_SLC12A8	+
295	chr3	129693450	129693571	TRH	+
296	chr3	137483866	137484158	SOX14	+
297	chr3	138658505	138658705	PIK3CB_FOXL2	+
298	chr3	138658926	138659107	PIK3CB_FOXL2	+
299	chr3	147109864	147110368	PLSCR1_ZIC4	-
300	chr3	157812116	157812476	VEPH1_SHOX2	+
301	chr3	171175949	171176142	SLC2A2_TNIK	+
302	chr3	171176243	171176245	SLC2A2_TNIK	+
303	chr3	188012869	188012956	TPRG1_LPP	+
304	chr3	192125798	192126293	FGF12	+
305	chr3	196367613	196367833	NRROS_CEP19	+
306	chr3	196387610	196387860	NRROS_CEP19	+
307	chr3	50378042	50378201	RASSF1A	-
308	chr4	3312001	3312113	RGS12	+
309	chr4	8582130	8582490	GPR78	+
310	chr4	8859833	8860023	HMX1_CPZ	+
311	chr4	8863184	8863458	HMX1_CPZ	+
312	chr4	13543524	13543881	RAB28_NKX3-2	+
313	chr4	24801661	24802067	SOD3_LGI2	+
314	chr4	38673052	38673145	KLF3_TLR10	+
315	chr4	38673288	38673290	KLF3_TLR10	+
316	chr4	44449524	44449797	KCTD8	+
317	chr4	57521296	57522019	HOPX_ARL9	+
318	chr4	74864253	74864404	CXCL5	+
319	chr4	94755793	94756007	SMARCAD1_ATOH1	+
320	chr4	111534015	111534312	PITX2_ENPEP	+
321	chr4	140656921	140657069	MGST2_MAML3	+
322	chr4	147559986	147560488	POU4F2	+
323	chr4	154709491	154709790	SFRP2	+

324	chr4	155664045	155664267	LRAT	+
325	chr4	158141376	158141695	GRIA2	+
326	chr4	190940246	190940450	FRG2_FRG1	+
327	chr4	81952348	81952412	BMP3	+
328	chr4	154710416	154710538	SFRP2	-
329	chr4	84035862	84035983	PLAC8	-
330	chr4	81187783	81187912	FGF5	+
331	chr5	1876285	1876484	IRX4_NDUFS6	+
332	chr5	1877873	1878377	IRX4_NDUFS6	+
333	chr5	2149463	2149653	IRX4_IRX2	+
334	chr5	5140527	5140709	ADAMTS16	+
335	chr5	16179957	16180455	MARCH11	-
336	chr5	40681168	40681354	PTGER4_PRKAA1	+
337	chr5	72594785	72594977	TMEM174_FOXD1	+
338	chr5	72715324	72715515	FOXD1_TMEM174	+
339	chr5	76249400	76249811	AGGF1_CRHBP	+
340	chr5	134374663	134374869	PITX1	-
341	chr5	140800861	140801091	PCDHGA11	+
342	chr5	140892821	140893043	PCDHGC5_DIAPH1	+
343	chr5	153862052	153862397	HAND1	-
344	chr5	168307143	168307354	PANK3_SLIT3	+
345	chr5	172672913	172673044	NKX2-5_STC2	+
346	chr5	177411394	177411831	PROP1_B4GALT7	+
347	chr5	178003723	178004060	PHYKPL_COL23A1	+
348	chr5	179894246	179894413	GFPT2_CNOT6	+
349	chr5	180486231	180486766	BTNL9_OR2V1	+
350	chr5	112073421	112073518	APC	+
351	chr5	115152460	115152575	CDO1	-
352	chr6	1378651	1378856	FOXF2_FOXQ1	+
353	chr6	6003907	6004299	NRN1_FARS2	-
354	chr6	10417573	10417778	TFAP2A	-
355	chr6	10421371	10421804	GCNT2_TFAP2A	+
356	chr6	19691729	19692173	ID4	+
357	chr6	30131360	30131614	TRIM10_TRIM15	-
358	chr6	32157425	32157687	PBX2	+
359	chr6	36253025	36253261	PNPLA1_ETV7	+
360	chr6	38683076	38683301	GLO1_DNAH8	+
361	chr6	41341406	41341751	FOXP4_NCR2	+
362	chr6	41528448	41528958	MDFI_FOXP4	+
363	chr6	42072032	42072648	GUCA1A_TAF8	+
364	chr6	50818148	50818365	TFAP2B	+
365	chr6	56716258	56716537	DST_KIAA1586	+
366	chr6	85476087	85476330	TBX18	-
367	chr6	85477013	85477284	TBX18	-
368	chr6	106433875	106434297	PREP_PRDM1	+
369	chr6	137814472	137814861	OLIG3	+
370	chr6	143234754	143234818	HIVEP2_GPR126	+
371	chr6	143234931	143235067	HIVEP2_GPR126	+
372	chr6	151004536	151004727	MTHFD1L_PLEKHG1	+
373	chr6	163817971	163818242	PARK2_QKI	+
374	chr6	170494223	170494454	DLL1_C6orf70	+
375	chr6	3247630	3247751	chr6:3	+
376	chr7	391393	391545	PDGFA_FAM20C	+
377	chr7	641090	641403	PDGFA_PRKAR1B	+
378	chr7	1263580	1263840	ZFAND2A_UNCX	+
379	chr7	4859593	4859779	PAPOLB_AP5Z1	+
380	chr7	27196417	27196625	HOXA7	-
381	chr7	27206024	27206238	HOXA9	-
382	chr7	27260129	27260467	EVX1_HOXA13	+
383	chr7	27283472	27283676	EVX1_HIBADH	+
384	chr7	29603208	29603408	PRR15	+
385	chr7	29605641	29606290	WIPF3_PRR15	+
386	chr7	35293620	35293984	TBX20	-
387	chr7	35297390	35297595	TBX20	-
388	chr7	55248872	55249088	LANCL2_EGFR	+

389	chr7	67016137	67016255	TYW1	+
390	chr7	70111546	70111758	WBSCR17	+
391	chr7	71802022	71802283	CALN1	-
392	chr7	97361441	97361635	TAC1	+
393	chr7	97361747	97361815	TAC1	+
394	chr7	121950117	121950351	FEZF1_RNF133	+
395	chr7	121950621	121950800	FEZF1_RNF133	+
396	chr7	127991841	127992012	RBM28_PRRT4	+
397	chr7	127992202	127992439	RBM28_PRRT4	+
398	chr7	150655279	150655530	KCNH2_AOC1	-
399	chr7	154409595	154409721	PAXIP1_DPP6	+
400	chr7	156400843	156401092	SHH_C7orf13	+
401	chr7	156798401	156798722	MNX1_NOM1	-
402	chr7	156810853	156811073	UBE3C_MNX1	+
403	chr7	156811251	156811441	UBE3C_MNX1	+
404	chr7	157478278	157478491	DNAJB6_PTPRN2	+
405	chr7	97361294	97361446	TAC1	+
406	chr7	27135828	27135898	HOXA1	-
407	chr7	50343867	50343961	IKZF1	+
408	chr8	686980	687150	DLGAP2_TDRP	+
409	chr8	687364	687587	DLGAP2_TDRP	+
410	chr8	3549494	3549784	NONE	+
411	chr8	10588882	10589207	SOX7	-
412	chr8	20375577	20375803	LZTS1	+
413	chr8	22876025	22876275	RHOBTB2_TNFRSF10B	+
414	chr8	23563997	23564326	NKX2-6	-
415	chr8	38757725	38758112	PLEKHA2	+
416	chr8	55370708	55370863	SOX17	+
417	chr8	55379519	55379958	RP1_SOX17	+
418	chr8	55382639	55382877	RP1_SOX17	+
419	chr8	56901630	56901792	RPS20_LYN	+
420	chr8	70946890	70947123	SLCO5A1_PRDM14	+
421	chr8	70984269	70984560	PRDM14	-
422	chr8	92607187	92607387	SLC26A7_RUNX1T1	+
423	chr8	97171939	97172197	GDF6	+
424	chr8	99986545	99987001	VPS13B_OSR2	+
425	chr8	116679722	116679936	TRPS1	+
426	chr8	143667537	143667764	ARC_BAI1	+
427	chr8	145105504	145106417	OPLAH_SPATC1	+
428	chr8	97506250	97506398	SDC2	+
429	chr8	41166232	41166296	SFRP1	+
430	chr8	55370371	55370440	SOX17	+
431	chr8	124332801	124332922	ATAD2	+
432	chr8	20375518	20375639	ch8:20	+
433	chr9	1042341	1042870	DMRT2_DMRT3	+
434	chr9	13323138	13323350	MPDZ_NFIB	+
435	chr9	14346833	14347127	NFIB_ZDHHC21	+
436	chr9	19788614	19788770	SLC24A2	-
437	chr9	19789005	19789196	SLC24A2	-
438	chr9	27690516	27690698	C9orf72_LINGO2	+
439	chr9	36986083	36986751	PAX5_MELK	+
440	chr9	36986898	36987141	PAX5_MELK	+
441	chr9	100616259	100616670	FOXE1	+
442	chr9	120507170	120507467	TLR4	+
443	chr9	126778344	126778602	NEK6_LHX2	+
444	chr9	127265692	127265900	NR5A1_GPR144	+
445	chr9	132650647	132650877	USP20_FNBP1	+
446	chr9	132651024	132651124	USP20_FNBP1	+
447	chr9	133771498	133771758	QRFP	+
448	chr9	135462414	135462593	GTF3C4_BARHL1	+
449	chr9	139428562	139428679	SEC16A_NOTCH1	+
450	chr9	21974677	21974779	CDKN2A	+
