# Supplementary material for: Liquid biopsy uncovers distinct patterns of DNA methylation and copy number changes in NSCLC patients with different EGFR-TKI resistant mutations
Source: Sci Rep. 2021 Aug 12;11:16436. doi: 10.1038/s41598-021-95985-6 (PMC8361064; doi:10.1038/s41598-021-95985-6)
Supplement: Supplementary file 9 — Supplementary Table S3. [file 41598_2021_95985_MOESM9_ESM.pdf]

Liquid biopsy uncovers distinct patterns of DNA methylation and copy number changes in NSCLC patients with different EGFR-TKI resistant mutations

Hoai-Nghia Nguyen, Ngoc-Phuong Thi Cao, Thien-Chi Van Nguyen, Khang Nguyen Duy Le, Dat Thanh Nguyen, Quynh-Tho Thi Nguyen, Thai-Hoa Thi Nguyen, Chu Van Nguyen, Ha Thu Le, Mai-Lan Thi Nguyen, Trieu Vu Nguyen, Vu Uyen Tran, Bac An Luong, Le Gia Hoang Le, Quoc Chuong Ho, Hong-Anh Thi Pham, Binh Thanh Vo, Luan Thanh Nguyen, Anh-Thu Huynh Dang, Sinh Duy Nguyen, Duc Minh Do, Thanh-Thuy Thi Do, Anh Vu Hoang, Kiet Truong Dinh, Minh-Duy Phan, Hoa Giang, Le Son Tran

Table S3: Differentially methylated regions between on-target and off-target resistance mutations, using Wilcoxon Signed Rank test

	chrom	start	end	Associated gene	strand	region size	wilcox_pval	log2FC	fdr
1	chr12	113901942	113901976	LHX5_SDSL	+	113	0.023	6.016	0.040
2	chr5	40681168	40681354	PTGER4_PRKAA1	+	336	0.001	5.066	0.004
3	chr12	6664804	6664910	IFFO1	-	98	0.001	5.065	0.004
4	chr1	48173969	48174156	FOXD2_TRABD2B	+	13	0.001	4.615	0.004
5	chr17	37321625	37321818	PLXDC1_ARL5C	+	180	0.001	4.611	0.004
6	chr22	19754706	19754971	TBX1_C22orf29	+	281	0.001	4.475	0.004
7	chr4	57521296	57522019	HOPX_ARL9	+	317	0.001	4.377	0.004
8	chr3	138658505	138658705	PIK3CB_FOXL2	+	297	0.001	4.329	0.004
9	chr12	6665056	6665530	IFFO1	-	99	0.003	4.238	0.010
10	chr12	25055818	25056391	BCAT1	-	101	0.014	4.126	0.027
11	chr1	29586187	29586646	PTPRU	+	10	0.001	4.120	0.004
12	chr2	73147404	73148014	EMX1_SFXN5	+	234	0.001	3.998	0.004
13	chr4	8859833	8860023	HMX1_CPZ	+	310	0.027	3.992	0.046
14	chr12	6664346	6664537	IFFO1	-	97	0.001	3.975	0.004
15	chr10	123923130	123923372	PLEKHA1_TACC2	+	64	0.001	3.965	0.004
16	chr5	76249400	76249811	AGGF1_CRHBP	+	339	0.002	3.927	0.007
17	chr17	59482165	59482348	TBX4_TBX2	+	187	0.001	3.794	0.004
18	chr6	42072032	42072648	GUCA1A_TAF8	+	363	0.001	3.743	0.004
19	chr20	55965076	55965310	RBM38	+	274	0.005	3.726	0.011
20	chr6	6003907	6004299	NRN1_FARS2	-	353	0.001	3.703	0.004
21	chr8	70984269	70984560	PRDM14	-	422	0.001	3.700	0.004
22	chr17	73749586	73749805	GALK1_ITGB4	+	194	0.005	3.699	0.011
23	chr2	106681891	106682238	C2orf40	+	242	0.001	3.695	0.004
24	chr5	115152460	115152575	CDO1	-	351	0.001	3.690	0.004
25	chr12	113901278	113901427	LHX5_SDSL	+	112	0.008	3.620	0.017
26	chr7	156798401	156798722	MNX1_NOM1	-	402	0.001	3.564	0.004
27	chr10	8094211	8094348	GATA3	+	49	0.001	3.552	0.004
28	chr3	124860393	124860960	HEG1_SLC12A8	+	294	0.001	3.543	0.004
29	chr2	171679590	171679872	GORASP2_GAD1	+	249	0.014	3.529	0.027
30	chr17	46621708	46621880	HOXB1_HOXB2	+	181	0.008	3.522	0.017
31	chr4	24801661	24802067	SOD3_LGI2	+	313	0.001	3.512	0.004
32	chr4	140656921	140657069	MGST2_MAML3	+	321	0.002	3.459	0.007
33	chr2	233925000	233925347	INPP5D	+	266	0.002	3.424	0.007
34	chr22	50987269	50987506	TYMP_SYCE3	+	289	0.001	3.412	0.004
35	chr5	172672913	172673044	NKX2-5_STC2	+	345	0.022	3.397	0.039
36	chr7	29605641	29606290	WIPF3_PRR15	+	386	0.001	3.386	0.004
37	chr19	46997011	46997223	CCDC8_PPP5D1	+	214	0.002	3.376	0.007
38	chr6	41528448	41528958	MDFI_FOXP4	+	362	0.001	3.367	0.004
39	chr10	94822336	94822766	CYP26A1_CYP26C1	+	57	0.001	3.365	0.004
40	chr1	248020558	248020995	TRIM58	+	44	0.008	3.322	0.017
41	chr7	27260129	27260467	EVX1_HOXA13	+	383	0.001	3.317	0.004
42	chr3	129693450	129693571	TRH	+	295	0.002	3.313	0.007
43	chr7	27135828	27135898	HOXA1	-	407	0.026	3.311	0.045
44	chr3	196367613	196367833	NRROS_CEP19	+	305	0.001	3.307	0.004
45	chr16	86612922	86613063	FOXL1	+	172	0.001	3.282	0.004
46	chr8	55370708	55370863	SOX17	+	417	0.002	3.274	0.007
47	chr21	38069496	38069867	SIM2	+	277	0.001	3.243	0.004
48	chr12	6184401	6184680	ANO2_VWF	+	96	0.001	3.223	0.004
49	chr11	131780423	131780494	OPCML	+	95	0.017	3.215	0.033
50	chr6	106433875	106434297	PREP_PRDM1	+	368	0.001	3.215	0.004
51	chr11	76750654	76750944	CAPN5_B3GNT6	+	91	0.001	3.214	0.004
52	chr6	56716258	56716537	DST_KIAA1586	+	365	0.014	3.171	0.027
53	chr1	119532693	119532983	TBX15	-	28	0.001	3.142	0.004
54	chr2	74726391	74726810	MRPL53_LBX2	+	235	0.001	3.133	0.004
55	chr3	138658926	138659107	PIK3CB_FOXL2	+	298	0.001	3.130	0.004
56	chr3	171175949	171176142	SLC2A2_TNIK	+	301	0.001	3.084	0.004
57	chr17	46832289	46832580	HOXB13_TTLL6	+	183	0.002	3.074	0.007
58	chr10	94828102	94828377	CYP26A1_CYP26C1	+	58	0.008	3.071	0.017
59	chr2	66666351	66666865	ETAA1_MEIS1	+	232	0.001	3.027	0.004
60	chr7	27206024	27206238	HOXA9	-	382	0.001	3.023	0.004
61	chr8	145105504	145106417	OPLAH_SPATC1	+	428	0.002	3.020	0.007
62	chr9	1042341	1042870	DMRT2_DMRT3	+	434	0.001	3.009	0.004
63	chr12	124941685	124942045	NCOR2_ZNF664	+	119	0.001	2.968	0.004
64	chr7	391393	391545	PDGFA_FAM20C	+	377	0.001	2.957	0.004
65	chr8	99986545	99987001	VPS13B_OSR2	+	425	0.005	2.957	0.011
66	chr7	121950117	121950351	FEZF1_RNF133	+	395	0.001	2.955	0.004
67	chr11	76033076	76033392	WNT11_PRKRIR	+	90	0.005	2.952	0.011
68	chr6	38683076	38683301	GLO1_DNAH8	+	360	0.001	2.943	0.004
69	chr15	41795000	41795251	ITPKA_LTK	+	158	0.001	2.918	0.004
70	chr6	10421371	10421804	GCNT2_TFAP2A	+	355	0.001	2.908	0.004
71	chr1	119526985	119527255	SPAG17_TBX15	+	27	0.001	2.893	0.004
72	chr2	223163710	223163894	PAX3	-	265	0.001	2.849	0.004
73	chr17	35299775	35299991	AATF_LHX1	+	179	0.001	2.840	0.004
74	chr6	151004536	151004727	MTHFD1L_PLEKHG1	+	372	0.001	2.831	0.004
75	chr10	129534706	129534913	FOXI2	+	69	0.002	2.828	0.007
76	chr15	76639691	76639823	ISL2_SCAPER	+	162	0.001	2.816	0.004
77	chr14	57275767	57276040	OTX2_TMEM260	-	143	0.001	2.808	0.004
78	chr10	17496615	17496781	ST8SIA6	-	78	0.008	2.792	0.017
79	chr13	25320074	25320295	RNF17_ATP12A	+	125	0.022	2.788	0.039
80	chr8	55370371	55370440	SOX17	+	431	0.022	2.784	0.039
81	chr22	19753101	19753479	TBX1_C22orf29	+	280	0.001	2.770	0.004
82	chr2	114034363	114034535	PAX8_PSD4	+	244	0.003	2.761	0.010
83	chr4	13543524	13543881	RAB28_NKX3-2	+	312	0.022	2.731	0.039
84	chr1	221050372	221050608	HLX	+	39	0.001	2.726	0.004

85	chr1	145562790	145563083	PIAS3_ITGA10	+	31	0.002	2.724	0.007
86	chr7	35297390	35297595	TBX20	-	388	0.001	2.721	0.004
87	chr13	100608122	100608388	ZIC5_CLYBL	+	129	0.014	2.716	0.027
88	chr1	63785840	63786084	FOXD3	+	17	0.001	2.695	0.004
89	chr11	125036382	125036637	PKNOX2_FEZ1	-	93	0.008	2.695	0.017
90	chr12	33592318	33592537	SYT10	-	102	0.022	2.684	0.039
91	chr10	102899822	102900142	TLX1_LBX1	+	60	0.001	2.645	0.004
92	chr10	124905496	124906129	HMX2	+	66	0.002	2.605	0.007
93	chr16	11327008	11327232	SOCS1_CIITA	+	164	0.002	2.600	0.007
94	chr16	86613195	86613349	FOXL1_FBXO31	+	173	0.002	2.600	0.007
95	chr3	171176243	171176245	SLC2A2_TNIK	+	302	0.018	2.595	0.034
96	chr2	176947022	176947263	KIAA1715_HOXD10	+	252	0.001	2.581	0.004
97	chr12	54441275	54441495	HOXC4_HOXC5	+	104	0.001	2.570	0.004
98	chr8	55379519	55379958	RP1_SOX17	+	418	0.001	2.560	0.004
99	chr2	176987302	176987846	HOXD9	+	256	0.001	2.556	0.004
100	chr7	1263580	1263840	ZFAND2A_UNCX	+	379	0.001	2.534	0.004
101	chr2	220313201	220313603	GMPPA_SPEG	+	263	0.001	2.510	0.004
102	chr2	30453574	30453794	LBH	+	224	0.001	2.499	0.004
103	chr17	59532094	59532280	TBX4	+	189	0.001	2.487	0.004
104	chr6	19691729	19692173	ID4	+	356	0.001	2.478	0.004
105	chr1	110626174	110626397	UBL4B_ALX3	+	23	0.001	2.455	0.004
106	chr12	114886354	114886619	TBX5_TBX3	+	117	0.005	2.453	0.011
107	chr2	45231696	45231832	SIX2_SIX3	+	229	0.001	2.444	0.004
108	chr15	41787837	41788308	ITPKA_LTK	+	156	0.002	2.434	0.007
109	chr5	140892821	140893043	PCDHGC5_DIAPH1	+	342	0.001	2.428	0.004
110	chr17	77789574	77789743	CBX8_CBX4	+	197	0.001	2.417	0.004
111	chr1	221050087	221050260	HLX	+	38	0.001	2.389	0.004
112	chr1	50884549	50885150	DMRTA2_ELAVL4	+	15	0.022	2.384	0.039
113	chr17	56405881	56406036	BZRAP1	+	186	0.001	2.383	0.004
114	chr16	88769853	88770135	CTU2_RNF166	+	174	0.001	2.371	0.004
115	chr10	126135970	126136163	OAT_NKX1-2	+	68	0.002	2.341	0.007
116	chr6	41341406	41341751	FOXP4_NCR2	+	361	0.001	2.317	0.004
117	chr5	153862052	153862397	HAND1	-	343	0.001	2.313	0.004
118	chr14	57264957	57265561	OTX2_TMEM260	-	142	0.001	2.310	0.004
119	chr1	119535682	119535909	TBX15	-	29	0.001	2.305	0.004
120	chr2	172965543	172965651	DLX2_DLX1	+	250	0.005	2.305	0.011
121	chr9	126778344	126778602	NEK6_LHX2	+	444	0.001	2.305	0.004
122	chr11	31837358	31837807	PAX6_ELP4	-	85	0.008	2.304	0.017
123	chr6	163817971	163818242	PARK2_QKI	+	373	0.008	2.299	0.017
124	chr8	116679722	116679936	TRPS1	+	426	0.008	2.261	0.017
125	chr20	55202127	55202359	TFAP2C	+	273	0.001	2.257	0.004
126	chr2	113931517	113931566	PSD4	+	243	0.001	2.251	0.004
127	chr13	100649566	100649848	PCCA_ZIC2	+	130	0.001	2.244	0.004
128	chr15	53083366	53083605	ONECUT1	-	160	0.001	2.242	0.004
129	chr2	45227751	45228030	SIX2_SIX3	+	228	0.001	2.213	0.004
130	chr2	177024277	177024416	HOXD1_HOXD4	+	260	0.001	2.205	0.004
131	chr2	176956534	176956707	HOXD10_HOXD11_	+	253	0.005	2.200	0.011
132	chr2	176964833	176965036	HOXD10_HOXD11_HOXD12	+	254	0.022	2.197	0.039
133	chr20	39597821	39598025	MAFB_TOP1	+	271	0.001	2.194	0.004
134	chr14	55243068	55243425	GCH1_SAMD4A	+	140	0.008	2.169	0.017
135	chr1	50885338	50885470	DMRTA2_ELAVL4	+	16	0.005	2.161	0.011
136	chr13	25320419	25320698	RNF17_ATP12A	+	126	0.005	2.152	0.011
137	chr12	114840799	114841333	RBM19_TBX5	+	116	0.008	2.151	0.017
138	chr13	53313409	53313622	LECT1	+	128	0.005	2.123	0.011
139	chr2	19549979	19550627	NT5C1B-RDH14_OSR1	+	223	0.014	2.118	0.027
140	chr5	1877873	1878377	IRX4_NDUFS6	+	332	0.005	2.102	0.011
141	chr17	48546637	48546885	CHAD	-	184	0.005	2.093	0.011
142	chr16	86612367	86612598	FOXL1	+	171	0.005	2.092	0.011
143	chr1	111813487	111813699	CHIA_CHI3L2	+	24	0.001	2.088	0.004
144	chr2	177017172	177017390	HOXD1_HOXD4	+	259	0.014	2.071	0.027
145	chr10	7449724	7449927	PRKCQ_SFMBT2	+	47	0.014	2.069	0.027
146	chr11	636846	637203	DRD4	+	81	0.005	2.068	0.011
147	chr14	21093698	21093907	RNASE12_OR6S1	+	139	0.008	2.061	0.017
148	chr11	62211803	62212115	SCGB1A1_AHNAK	+	88	0.005	2.036	0.011
149	chr1	110612460	110612814	ALX3	+	22	0.001	2.029	0.004
150	chr1	119522367	119522549	SPAG17_TBX15	+	26	0.001	2.028	0.004
151	chr4	190940246	190940450	FRG2_FRG1	+	326	0.002	2.019	0.007
152	chr2	176969341	176969613	HOXD10_HOXD11	+	255	0.001	2.000	0.004
153	chr4	155664045	155664267	LRAT	+	324	0.002	1.970	0.007
154	chr6	10417573	10417778	TFAP2A	-	354	0.022	1.956	0.039
155	chr7	27283472	27283676	EVX1_HIBADH	+	384	0.022	1.949	0.039
156	chr4	94755793	94756007	SMARCAD1_ATOH1	+	319	0.001	1.946	0.004
157	chr17	55520553	55520788	MSI2_ENSG00000166329	+	185	0.001	1.936	0.004
158	chr10	103044094	103044401	BTRC_LBX1	+	61	0.002	1.931	0.007
159	chr7	156811251	156811441	UBE3C_MNX1	+	404	0.008	1.924	0.017
160	chr5	140800861	140801091	PCDHGA11	+	341	0.001	1.923	0.004
161	chr14	105750446	105750677	PACS2_BTBD6	+	151	0.001	1.919	0.004
162	chr4	111534015	111534312	PITX2_ENPEP	+	320	0.014	1.909	0.027
163	chr17	73483971	73483973	CASKIN2_KIAA0195	+	191	0.025	1.899	0.043
164	chr8	22876025	22876275	RHOBTB2_TNFRSF10B	+	414	0.014	1.896	0.027
165	chr5	134374663	134374869	PITX1	-	340	0.001	1.878	0.004
166	chr2	105458972	105459192	POU3F3	+	239	0.008	1.877	0.017
167	chr2	176988063	176988243	HOXD8_HOXD9	+	257	0.022	1.877	0.039
168	chr2	45028955	45029236	SIX3_CAMKMT	+	227	0.001	1.846	0.004
169	chr10	74078123	74078156	DNAJB12_DDIT4	+	54	0.002	1.837	0.007
170	chr16	70771557	70771814	MTSS1L_VAC14	+	169	0.005	1.825	0.011
171	chr15	45427308	45427482	DUOX1_SHF	+	159	0.001	1.821	0.004

172	chr2	223163256	223163509	PAX3	-	264	0.005	1.812	0.011
173	chr2	99439492	99439644	MGAT4A_TSGA10	+	238	0.005	1.786	0.011
174	chr2	85811470	85811855	VAMP5	+	236	0.008	1.785	0.017
175	chr8	70946890	70947123	SLCO5A1_PRDM14	+	421	0.005	1.765	0.011
176	chr9	132650647	132650877	USP20_FNBP1	+	446	0.014	1.754	0.027
177	chr7	150655279	150655530	KCNH2_AOC1	-	399	0.002	1.747	0.007
178	chr9	36986898	36987141	PAX5_MELK	+	441	0.001	1.706	0.004
179	chr13	51417487	51417687	RNASEH2B_DLEU1	+	127	0.008	1.705	0.017
180	chr3	157812116	157812476	VEPH1_SHOX2	+	300	0.001	1.698	0.004
181	chr8	686980	687150	DLGAP2_TDRP	+	409	0.002	1.646	0.007
182	chr7	157478278	157478491	DNAJB6_PTPRN2	+	405	0.005	1.637	0.011
183	chr7	156810853	156811073	UBE3C_MNX1	+	403	0.005	1.634	0.011
184	chr1	2705890	2706174	ACTRT2_MMEL1	+	3	0.014	1.626	0.027
185	chr8	38757725	38758112	PLEKHA2	+	416	0.005	1.618	0.011
186	chr14	97499574	97500077	VRK1	+	149	0.001	1.573	0.004
187	chr6	36253025	36253261	PNPLA1_ETV7	+	359	0.008	1.528	0.017
188	chr7	641090	641403	PDGFA_PRKAR1B	+	378	0.014	1.503	0.027
189	chr1	15672438	15672742	EFHD2_TMEM51	+	9	0.001	1.496	0.004
190	chr1	2706288	2706691	ACTRT2_MMEL1	+	4	0.001	1.482	0.004
191	chr1	161038973	161039034	ARHGAP30	+	36	0.012	1.475	0.026
192	chr13	112547627	112548039	SOX1_TEX29	+	134	0.008	1.457	0.017
193	chr21	38068722	38068902	SIM2	+	276	0.001	1.451	0.004
194	chr5	177411394	177411831	PROP1_B4GALT7	+	346	0.022	1.432	0.039
195	chr9	127265692	127265900	NR5A1_GPR144	+	445	0.005	1.378	0.011
196	chr2	10471576	10471983	HPCAL1_ODC1	+	222	0.001	1.372	0.004
197	chr8	10588882	10589207	SOX7	-	412	0.008	1.370	0.017
198	chr1	155265002	155265487	PKLR_HCN3	+	32	0.001	1.286	0.004
199	chr6	85477013	85477284	TBX18	-	367	0.002	1.245	0.007
200	chr1	50881990	50882155	DMRTA2_ELAVL4	+	14	0.022	1.204	0.039
201	chr4	84035862	84035983	PLAC8	-	329	0.008	1.169	0.017
202	chr1	111813838	111814003	CHIA_CHI3L2	+	25	0.008	1.160	0.017
203	chr22	51016357	51016608	CPT1B	-	290	0.002	0.921	0.007
204	chr10	94451964	94452121	EXOC6_HHEX	+	56	0.002	0.829	0.007
205	chr9	139428562	139428679	SEC16A_NOTCH1	+	450	0.014	0.445	0.027
206	chr17	73484081	73484191	CASKIN2_KIAA0195	+	192	0.005	0.315	0.011
207	chr15	41793266	41793500	ITPKA_LTK	+	157	0.005	0.291	0.011
208	chr17	78999556	78999742	BAIAP2_CHMP6	+	198	0.005	-0.088	0.011
209	chr7	70111546	70111758	WBSCR17	+	391	0.005	-0.100	0.011
210	chr11	61536887	61537195	MYRF_TMEM258	+	87	0.022	-0.123	0.039
211	chr18	76709408	76709588	SALL3	+	203	0.002	-0.159	0.007
212	chr3	52828531	52828707	ITIH3	+	292	0.022	-0.174	0.039
213	chr7	4859593	4859779	PAPOLB_AP5Z1	+	380	0.001	-0.184	0.004
214	chr2	86163782	86164042	ST3GAL5_POLR1A	+	237	0.022	-0.186	0.039
215	chr13	113436467	113436692	MCF2L_ATP11A	+	136	0.008	-0.209	0.017
216	chr4	38673288	38673290	KLF3_TLR10	+	315	0.008	-0.214	0.017
217	chr2	1036660	1036693	TPO_SNTG2	+	219	0.005	-0.233	0.011
218	chr10	131213445	131213769	MGMT	+	71	0.022	-0.240	0.039
219	chr19	641770	641986	FGF22_RNF126	+	204	0.001	-0.244	0.004
220	chr2	47270901	47270918	TTC7A_CALM2	+	230	0.014	-0.245	0.027
221	chr17	80847078	80847210	ZNF750_B3GNTL1	+	200	0.001	-0.251	0.004
222	chr1	37941178	37941397	ZC3H12A_MEAF6	+	11	0.005	-0.263	0.011
223	chr10	130732305	130732507	MKI67_MGMT	+	70	0.002	-0.265	0.007
224	chr22	46403725	46404141	PPARA_WNT7B	+	287	0.008	-0.285	0.017
225	chr1	6125059	6125309	KCNAB2_CHD5	+	5	0.002	-0.286	0.007
226	chr2	1036555	1036557	TPO_SNTG2	+	218	0.018	-0.289	0.035
227	chr12	122473180	122473464	MLXIP_BCL7A	+	118	0.003	-0.296	0.010
228	chr1	156092387	156092640	SEMA4A_LMNA	+	33	0.022	-0.297	0.039
229	chr2	47271039	47271132	TTC7A_CALM2	+	231	0.015	-0.303	0.029
230	chr17	77386138	77386283	RBFOX3_ENGASE	+	196	0.001	-0.307	0.004
231	chr17	25867745	25867753	LGALS9_KSR1	+	177	0.022	-0.321	0.039
232	chr16	57654201	57654538	GPR56_GPR114	+	168	0.001	-0.324	0.004
233	chr17	80846793	80846966	ZNF750_B3GNTL1	+	199	0.001	-0.324	0.004
234	chr22	26446247	26446382	SEZ6L_MYO18B	+	285	0.014	-0.339	0.027
235	chr8	143667537	143667764	ARC_BAI1	+	427	0.002	-0.348	0.007
236	chr1	7729026	7729031	VAMP3_CAMTA1	+	7	0.022	-0.357	0.039
237	chr3	196387610	196387860	NRROS_CEP19	+	306	0.001	-0.360	0.004
238	chr10	74210234	74210297	DNAJB12_MICU1	+	55	0.003	-0.366	0.010
239	chr8	3549494	3549784	NONE	+	411	0.001	-0.379	0.004
240	chr1	7728778	7728879	VAMP3_CAMTA1	+	6	0.022	-0.386	0.039
241	chr9	133771498	133771758	QRFP	+	448	0.002	-0.424	0.007
242	chr22	20267802	20267971	RTN4R_DGCR6L	+	282	0.008	-0.459	0.017
243	chr7	154409595	154409721	PAXIP1_DPP6	+	400	0.005	-0.464	0.011
244	chr14	60207779	60207902	RTN1_JKAMP	+	144	0.014	-0.467	0.027
245	chr11	1331724	1331858	TOLLIP	-	82	0.022	-0.473	0.039
246	chr2	2120370	2120628	PXDN_MYT1L	+	220	0.001	-0.474	0.004
247	chr12	7072502	7072697	PHB2_PTPN6	+	100	0.008	-0.503	0.017
248	chr19	57587879	57587906	ZIM2_USP29	+	216	0.008	-0.504	0.017
249	chr6	170494223	170494454	DLL1_C6orf70	+	374	0.005	-0.509	0.011
250	chr1	1098979	1099212	C1orf159_TTLL10	+	1	0.001	-0.542	0.004
251	chr19	49238427	49238813	MAMSTR_RASIP1	+	215	0.002	-0.569	0.007
252	chr22	28195841	28196138	MN1	+	286	0.001	-0.570	0.004
253	chr14	93154069	93154333	LGMN_RIN3	+	147	0.001	-0.640	0.004
254	chr5	168307143	168307354	PANK3_SLIT3	+	344	0.008	-0.670	0.017
255	chr5	179894246	179894413	GFPT2_CNOT6	+	348	0.001	-0.693	0.004
256	chr14	77491953	77492311	IRF2BPL_VASH1	+	145	0.001	-0.721	0.004
257	chr14	93153542	93153863	LGMN_RIN3	+	146	0.001	-0.736	0.004
258	chr5	2149463	2149653	IRX4_IRX2	+	333	0.001	-0.864	0.004

259	chr7	67016137	67016255	TYW1	+	390	0.001	-0.896	0.004
260	chr10	3514754	3514827	PITRM1_KLF6	+	46	0.005	-1.605	0.011
