# Supplementary material for: Liquid biopsy uncovers distinct patterns of DNA methylation and copy number changes in NSCLC patients with different EGFR-TKI resistant mutations
Source: Sci Rep. 2021 Aug 12;11:16436. doi: 10.1038/s41598-021-95985-6 (PMC8361064; doi:10.1038/s41598-021-95985-6)
Supplement: Supplementary file 10 — Supplementary Table S4. [file 41598_2021_95985_MOESM10_ESM.pdf]

Liquid biopsy uncovers distinct patterns of DNA methylation and copy number changes in NSCLC patients with different EGFR-TKI resistant mutations

Hoai-Nghia Nguyen, Ngoc-Phuong Thi Cao, Thien-Chi Van Nguyen, Khang Nguyen Duy Le, Dat Thanh Nguyen, Quynh-Tho Thi Nguyen, Thai-Hoa Thi Nguyen, Chu Van Nguyen, Ha Thu Le, Mai-Lan Thi Nguyen, Trieu Vu Nguyen, Vu Uyen Tran, Bac An Luong, Le Gia Hoang Le, Quoc Chuong Ho, Hong-Anh Thi Pham, Binh Thanh Vo, Luan Thanh Nguyen, Anh-Thu Huynh Dang, Sinh Duy Nguyen, Duc Minh Do, Thanh-Thuy Thi Do, Anh Vu Hoang, Kiet Truong Dinh, Minh-Duy Phan, Hoa Giang, Le Son Tran

**Table S4**: list of genes associated with 5 significantly enriched pathways analysed by g:Profiler					
	**sources**				
	**KEGG**	**KEGG**	**REAC**	**REAC**	**REAC**
term_name	Signaling pathways regulating pluripotency of stem cells	Maturity onset diabetes of the young	TFAP2 (AP-2) family regulates transcription of other transcription factors	Activation of HOX genes during differentiation	Activation of anterior HOX genes in hindbrain development during early embryogenesis
term_id	KEGG:04550	KEGG:04950	REAC:R-HSA-8866906	REAC:R-HSA-5619507	REAC:R-HSA-5617472
adjusted_p_value	8.22833E-06	0.000305328	0.008212658	0.016298123	0.016298123
negative_log10_of_adjusted_p_value	5.084688284	3.515233145	2.085516262	1.787862412	1.787862412
term_size	143	26	4	120	120
query_size	133	133	182	182	182
intersection_size	14	6	3	10	10
effective_domain_size	8000	8000	10622	10622	10622
intersections	LHX5,PIK3CB,HOXB1,HOXA1,MEIS1, WNT11,ID4,TBX3,HAND1,PAX6, ONECUT1,HOXD1,SMARCAD1, WNT7B	MNX1,SLC2A2,PAX6,ONECUT1, PKLR,HHEX	TFAP2A,TFAP2C,PITX2	HOXB1,HOXB2,MEIS1,HOXC4,PAX6, HOXD1,HOXD4,MAFB,PAXIP1,CNOT6	HOXB1,HOXB2,MEIS1,HOXC4,PAX6, HOXD1,HOXD4,MAFB,PAXIP1,CNOT6
